# Uncovering a 500 million year old history and evidence of pseudogenization for TLR15

**DOI:** 10.3389/fimmu.2022.1020601

**Published:** 2022-12-20

**Authors:** Fabiana Neves, Antonio Muñoz-Mérida, André M. Machado, Tereza Almeida, Arnaud Gaigher, Pedro J. Esteves, L. Filipe C. Castro, Ana Veríssimo

**Affiliations:** ^1^ CIBIO‐InBIO, Research Center in Biodiversity and Genetic Resources, University of Porto, Vairão, Portugal; ^2^ BIOPOLIS Program in Genomics, Biodiversity and Land Planning, CIBIO, Vairão, Portugal; ^3^ Department of Biology, Faculty of Sciences, University of Porto, Porto, Portugal; ^4^ CIIMAR - Interdisciplinary Centre of Marine and Environmental Research, University of Porto, Matosinhos, Portugal; ^5^ Research Group for Evolutionary Immunogenomics, Max Planck Institute for Evolutionary Biology, Plön, Germany; ^6^ Research Unit for Evolutionary Immunogenomics, Department of Biology, University of Hamburg, Hamburg, Germany; ^7^ CITS - Center of Investigation in Health Technologies, CESPU, Gandra, Portugal

**Keywords:** Toll-like receptor 15, evolution, pseudogenization, negative selection, jawed vertebrates

## Abstract

**Introduction:**

Toll like receptors (TLRs) are at the front line of pathogen recognition and host immune response. Many TLR genes have been described to date with some being found across metazoans while others are restricted to specific lineages. A cryptic member of the TLR gene family, TLR15, has a unique phylogenetic distribution. Initially described in extant species of birds and reptiles, an ortholog has been reported for cartilaginous fish.

**Methods:**

Here, we significantly expanded the evolutionary analysis of TLR15 gene evolution, taking advantage of large genomic and transcriptomic resources available from different lineages of vertebrates. Additionally, we objectively search for TLR15 in lobe-finned and ray-finned fish, as well as in cartilaginous fish and jawless vertebrates.

**Results and discussion:**

We confirm the presence of TLR15 in early branching jawed vertebrates – the cartilaginous fish, as well as in basal Sarcopterygii – in lungfish. However, within cartilaginous fish, the gene is present in Holocephalans (all three families) but not in Elasmobranchs (its sister-lineage). Holocephalans have long TLR15 protein sequences that disrupt the typical TLR structure, and some species display a pseudogene sequence due to the presence of frameshift mutations and early stop codons. Additionally, TLR15 has low expression levels in holocephalans when compared with other TLR genes. In turn, lungfish also have long TLR15 protein sequences but the protein structure is not compromised. Finally, TLR15 presents several sites under negative selection. Overall, these results suggest that TLR15 is an ancient TLR gene and is experiencing ongoing pseudogenization in early-branching vertebrates.

## Introduction

The innate immune system is the first line of defense against invading pathogens, with a crucial role in establishing and shaping the adaptive immune response. Toll-like receptors (TLRs) are a major class of pattern recognition receptors (PRRs) able to recognize a wide variety of highly conserved pathogen-associated molecular patterns (PAMPs) and endogenous damage-associated molecular patterns (DAMPs), and promptly initiate an innate immune response (1). TLRs are considered the primary sensors of pathogens, being able to distinguish between self and non-self (1, 2). These type I transmembrane glycoproteins consist of an N-terminal extracellular ligand-binding domain containing a varying number of leucine-rich-repeat (LRR) motifs with a characteristic horseshoe-shaped solenoid structure, a single transmembrane (TM) region and a C-terminal intracellular toll-interleukin1 receptor (TIR) domain that mediates signaling (3, 4).

First identified in *Drosophila melanogaster* (5), TLRs are evolutionarily conserved in Metazoa (1). So far, 28 functional TLRs have been described in vertebrate species, with extensive gene repertoire variations between lineages (e.g. 21 in teleost fish and 10 in primates) (6, 7). The overall ectodomain architecture and phylogenetic criteria support a division into seven major subfamilies: TLR1, TLR3, TLR4, TLR5, TLR7, TLR11 and TLR13 (1, 3, 8). Previous works suggest that TLRs evolved by gene duplication (8, 9), a process considered the major driving force of evolutionary novelty, playing important roles in the evolution of vertebrate genomes (10, 11). As in several other multigene families, the evolutionary history of TLRs is marked by gene gain/loss events, with most vertebrate TLRs arising after the emergence of vertebrates and rapidly diversifying (6). Indeed, while TLR3 is the most conserved and ancient subfamily with no gene duplication events described, the TLR1 subfamily presents comparatively more gene gains (TLR6 and TLR10 in mammals, TLR1A, TLR1B, TLR2A, TLR2B, LR15, and TLR21 in birds, TLR18, TLR23, TLR25, and TLR27 in teleosts) and gene losses (TLR5, TLR8 and TLR9 in some birds, TLR15 in penguins and TLR23 in tetrapods) (6).

Recently, a novel gene lineage designated TLR15 was described as being unique to birds and some reptiles (12, 13), where it was recognized to participate in the viral and non-viral host immune response (6). Indeed, it has an important role in the immune response to different bacteria (14, 15), lysates from yeast (12) and also viruses (16, 17). Later, a putative TLR15 ortholog was also identified in a cartilaginous fish, the Australian ghost shark (*Callorhinchus milii*) (18). The reported presence of TLR15 in a cartilaginous fish was surprising since it suggested a much earlier origin than previously assumed, dating back to the ancestor of all jawed vertebrate > 450 million years ago (mya), but also implying secondary gene losses in many other vertebrate lineages (19). Despite being first described as a member of the TLR1 family (8), some authors have since proposed that TLR15 should be considered as a separate family. Indeed, TLR15 appears to have evolved independently from other TLR1 subfamily members (18, 20), and shows structural differences such as an intact asparagine ladder and an ectodomain with single-domain architecture, instead of the three-domain architecture shared by all TLR1 family members (3, 6).

Despite the importance of TLR genes in immune responses and their remarkable diversity, there is still a huge gap of knowledge on the evolutionary history of TLR genes across vertebrates and specifically in early-branching vertebrate lineages. Here we focus specifically on TLR15 to confirm its origin in a gnathostome ancestor (~450 mya) and survey its presence in an ample set of vertebrate lineages, making use of available genomic and transcriptomic resources. We also provide an analysis of the TLR15 evolution in vertebrates.

## Material and methods

### Bioinformatic searches

We used the previously described chicken and the Australian ghost shark (*C. milii*) TLR15 protein sequences (18) as queries to perform exhaustive blast searches on taxa representative of different vertebrate lineages, including mammals, reptiles/birds, amphibians, lungfishes, the coelacanth, ray-finned fish, cartilaginous fish (elasmobranchs and holocephalans), lampreys and hagfish genomes (**Supplementary Table 1**). Additionally, for holocephalans, searches were also performed in unpublished genomic databases (Castro et al. *in prep*) covering the three taxonomic orders from Chimaeriformes, namely Callorhinchidae (*Callorhinchus millii*), Chimaeridae (*Chimaera opalescens; Hydrolagus affinis*, *H. colliei*, and *H. mirabilis*) and Rhinochimaeridae (*Harriotta raleighana*). All the protein sequences retrieved (**Supplementary Figure 1**, **Supplementary Data 1**) were aligned using Multiple sequence Comparison by Log-Expectation (MUSCLE) as implemented in Geneious Prime (http://www.geneious.com), and the position of indels were adjusted manually. Sequence alignment of putatively functional proteins can be found in **Supplementary Figure 1**, while the alignment including putatively non-functional proteins, i.e., pseudogenes, can be found in **Supplementary Figures 2A, B**.

### Synteny analyses

**Figure 1:**
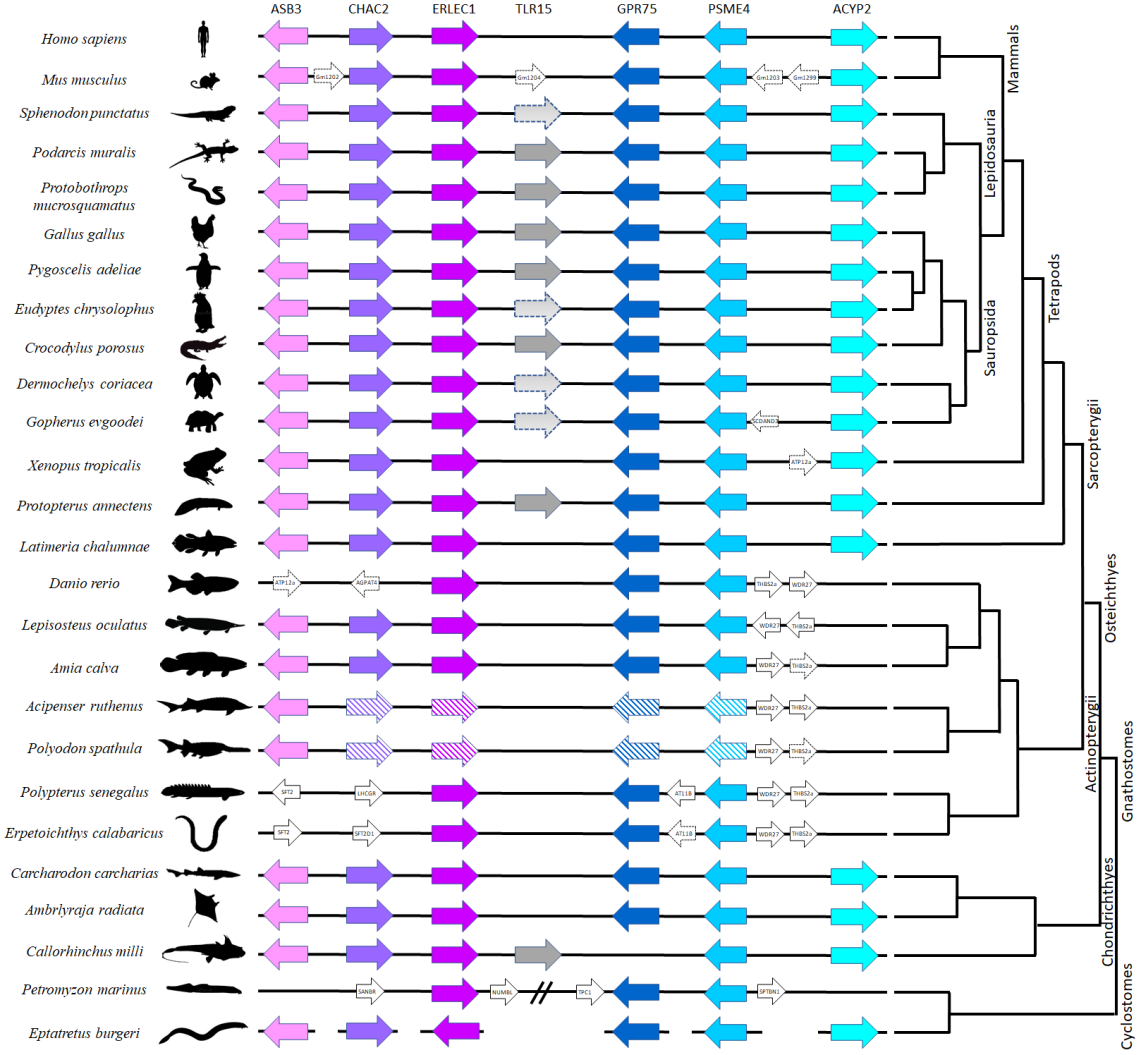
Comparative synteny analysis of TLR15 among representative vertebrate species. The same genes are represented in similar color across species. Arrows indicate the direction of translation. The line breaks indicate a larger distance between genes; the line gaps indicate that the genes are in separate chromosomes/Scaffolds and the genes with striped background are duplicated on different chromosomes (**Supplementary Table 4)**. The distances between genes are not scaled, and the corresponding chromosome locations are indicated in **Supplementary Table 4**.

The genomic region surrounding the TLR15 gene was surveyed for all neighboring genes (up to three genes upstream and downstream of TLR15) in several taxa representative of the different vertebrate lineages, using the gene annotations available on NCBI for each taxon. This analysis allowed comparisons of the gene composition and order across vertebrates and insights into the putative conserved TLR15 synteny reported previously (18). When any of the conserved syntenic genes were absent in some species, we conducted additional blastn and blastx searches (with default parameters) on publicly available and unpublished genomes (**Supplementary Table 1**). For those species in which no TLR15 ortholog was found, the genomic region between the two flanking genes common across vertebrates (ERLEC1 and GPR75) was subjected to blastx searches against NCBI non-redundant protein sequence database (nr). We performed this approach in different species of cartilaginous fishes, ray-finned fish, the coelacanth, lungfish, amphibians, turtles, crocodilians, birds, squamates, tuatara and mammals (**Supplementary Table 1**; **Figure 1**).

### Phylogenetic analysis

**Figure 2:**
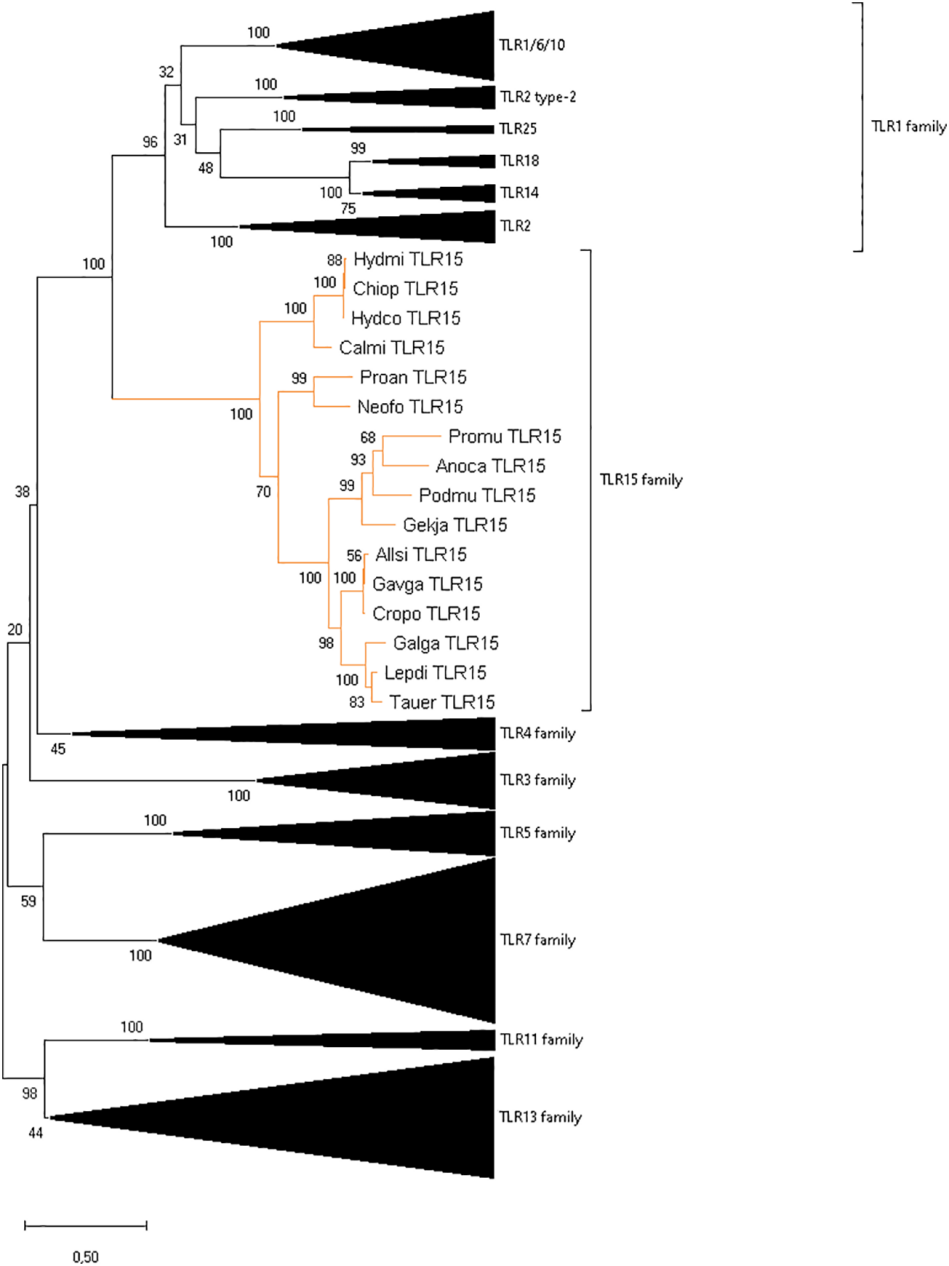
Reconstruction of the phylogeny of vertebrate TLR genes showing the division into eight families. The ML tree was built using 259 full length sequences representative of different vertebrate groups, the JTT+G+I amino acid substitution model, and mid-point rooting. The species abbreviations are the same as in **Supplementary Figure 1**.

To clarify the identification of TLR15 as an independent family, the full-length proteins (**Figure 2**) of vertebrate TLR members of all TLR families were used and aligned (data not shown) using MUSCLE (as described above). A phylogenetic reconstruction of vertebrate TLRs relationships was performed in MEGAX using the ML method, with JTT+G+I as the best-fit amino acid substitution model (determined by MEGAX using ML as statistical method), and mid-point rooting. All positions with less than 95% site coverage were eliminated (i.e., fewer than 5% alignment gaps, missing data, and ambiguous bases were allowed at any position) using the partial deletion option. The final dataset used 352 positions from a total of 1800 positions from 259 amino acid sequences.

### Protein structure modeling

Genomic DNA sequences corresponding to the putative TLR15 orthologs were used for prediction of intron-exon boundaries and domain structure, namely signal peptide, ectodomain (ECD), transmembrane region (TM) and Toll/interleukin-1 receptor (TIR) domain, using the SignalP6.0 server (https://services.healthtech.dtu.dk/service.php?SignalP) (21), SMART (http://smart.embl-heidelberg.de/) (22) and TMHMM (https://services.healthtech.dtu.dk/service.php?TMHMM-2.0) (23). Delimitation of LRR motifs was performed using different tools: LRRpredictor (https://lrrpredictor.biochim.ro/) (24), LRRsearch (http://lrrsearch.com/index.php?page=tool) (25) and Conserved Domain Database (www.ncbi.nlm.nih.gov/Structure/cdd/wrpsb.cgi) (26). At the time of manuscript preparation, the commonly used tool LRRfinder was not available due to technical issues. Thus, we adopted a conservative approach for LRR motif delimitation: only the motifs detected by at least 2 of the above tools were considered. Additionally, we used the I-TASSER webserver (https://zhanggroup.org/I-TASSER/) (27) to predict the three-dimensional (3D) structure of TLR15 in lungfish and holocephalans and the modeled structure was displayed by PyMol (Schrödinger, LLC).

### Residue analysis

The evolutionary dynamics of amino acid substitution among TLR15 proteins was estimated using the ConSurf algorithm (28). For this we used the TLR15 amino acid alignment (**Supplementary Figure 1**), and the chicken protein as query sequence. We allowed the algorithm to infer the phylogenetic tree using a maximum likelihood (ML) approach and the best evolutionary substitution model. The conservation scale retrieved is defined from the most variable positions to the most conserved positions (**Supplementary Figure 3**).

To validate the pseudogene allele retrieved for *H. affinis*, and to confirm that the large insertion present in holocephalans were indeed part of the transcript (and not an intron), we performed PCR amplification from cDNA of *H. affinis.* Thus, we designed the primers Forward: 5’ GGAATTCTAGCAACTGAGGAGAAAGAGG 3’ and Reverse: 5’ GAAAGGTCCAGAATTTCAAGAGAG 3’, and the PCR was made with the Multiplex PCR Kit (Qiagen, Hilden, Germany) according to the manufacturer ‘ s protocol. Sequencing was performed on an ABI PRISM 3100 Genetic Analyzer (PE Applied Biosystems) and PCR products were sequenced in both directions (**Supplementary Figure 2B**). Due to sample limitations, we were not able to corroborate by PCR the pseudogenization in *H. raleighana*. Additionally, to test if the larger insertions in holocephalans were part of the TLR15 gene or correspond to an intron, the gene annotation software AUGUSTUS (v. 3.3.2) (29) and GeneMark (v. 3.61) (30) were used to check for the presence of introns in the holocephalan TLR15 sequences. Results including start and stop codons, exons and introns were produced in gff3 format.

### Positive selection

The ratio (ω) of non-synonymous substitutions per non-synonymous sites (dN) over synonymous substitutions per synonymous sites (dS), dN/dS, was used to infer the selection pressures acting on TLR15 (Supplementary Tables 2 and 3). For this we used two ML frameworks, the CODEML program of Phylogenetic Analysis by Maximum Likelihood (PAML) 4.9 package (31, 32), and the HyPhy package implemented in the Datamonkey webserver (33, 34). In CODEML, a neighbour-joining tree of the TLR15 gene constructed in MEGAX (35) (with options: p-distances as the substitution model and complete deletion for gaps/missing data) was used as guide tree to compare the opposing site models M7 vs M8 using Likelihood Ratio Tests (LRT). While M7 (i.e. null model) assumes that ω ratios are distributed among sites according to a beta distribution allowing codons to evolve neutrally or under negative selection, M8 is an extension of the M7 model with an extra class of sites with an independent ω ratio freely estimated from the data allowing positive selection. Both, M7 and M8 models were compared by taking twice the difference in log likelihood between the two models, and the obtained value was assessed with a χ^2^ distribution (df = 2) to test the null model (p<0.05). Amino acids detected as under positive selection were identified using the Bayes Empirical Bayes (BEB) approach, with posterior probability > 95%. BEB is the preferred approach because it accounts for sampling errors in the ML (31, 32, 36–38).

In the datamonkey Web Server, all the methods can take recombination into account. Thus, prior to the selection analysis we used the GARD module (39) to screen our sequences for recombination breakpoints. Since recombination breakpoints were detected for the TLR15 gene, we used the partitioned dataset obtained in GARD as input for the selection models. The nucleotide sequences of the TLR15 were analyzed under four available models: Single Likelihood Ancestor Counting (SLAC), Fixed-Effect Likelihood (FEL), Mixed Effects Model of Evolution (MEME) and Fast Unconstrained Bayesian AppRoximation (FUBAR). The SLAC model is based on the reconstruction of ancestral sequences and counts the number of d_S_ and d_N_ changes at each codon position of the phylogeny (40). FEL estimates ratios of d_N_ to d_S_ changes for each site in an alignment (40). MEME detects sites evolving under positive selection under a proportion of branches (41). FUBAR detects site-specific selection assuming that the selection pressure for each site is constant along the entire phylogeny (42). For SLAC, FEL and MEME the p-value was set to ≤0.05, while for FUBAR we used a posterior probability ≥ 0.95. For a more conservative approach, and as used previously (43, 44), only sites detected to be under positive selection in more than one ML method were considered.

### Gene expression

**Figure 3:**
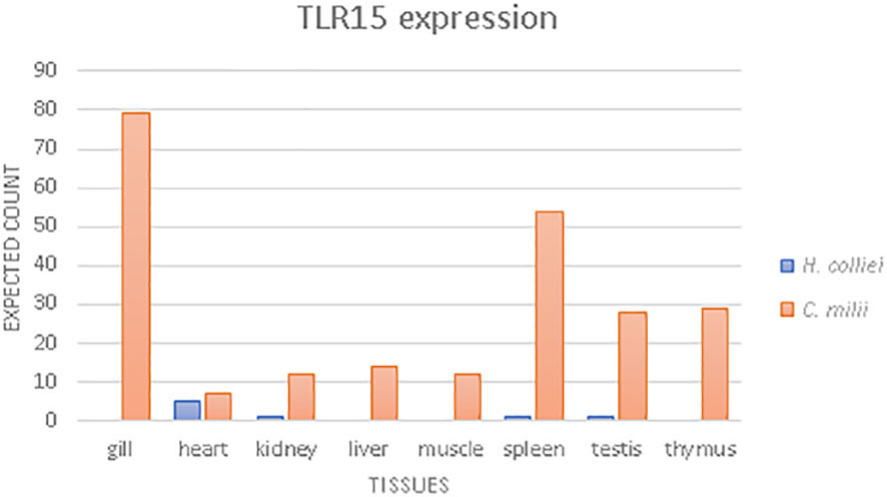
TLR15 gene expression analysis, based on the expected reads counts estimated with RSEM for Australian ghost shark (*C. milii*) and spotted ratfish (*H. colliei*).

Gene expression quantification was performed for TLR15 transcripts using bioinformatic mapping of the paired reads with RSEM (RNA-Seq by Expectation Maximization) (version 1.3.1.) by calling *rsem-prepare-reference* with specific parameter –bowtie2 and the *rsem-calculate-expression* with default parameters for paired-end reads (45). Read mapping was performed separately for *C. milli* and *H. colliei* for which RNAseq data was available from NCBI and from unpublished data (LFC Castro, *in prep*), using species-specific TLR15 transcripts as references (**Figure 3**). TLR2 and TLR3 gene expression were also estimated for each species (**Supplementary Figures 4, 5**, respectively), following the procedure described above, to allow insights into the relative expression levels of TLR15 compared to other constitutively expressed TLRs, and to ensure that the observed read counts were not due to unbalanced representation of genes in the dataset. The final read counts are in transcripts per million (TPM) and fragments per kilobase of transcript per million of fragments mapped (FPKM).

## Results

### TLR15 was present in the ancestor of jawed vertebrates

We found single-copy TLR15 gene orthologs in two jawed vertebrate lineages: holocephalans and lungfishes. The retrieved protein sequences share the conserved synteny (**Figure 1**) and cluster with other TLR15 members in a phylogenetic analysis (**Figure 2**). Among holocephalans, TLR15 gene orthologs were found in all species studied, covering the three taxonomic families included in the Order Chimaeriformes (**Supplementary Figures 1, 2**), namely Callorhinchidae (*Callorhinchus millii*), Chimaeridae (*Chimaera opalescens; Hydrolagus affinis*, *H. colliei*, and *H. mirabilis*) and Rhinochimaeridae (*Harriotta raleighana*) (accession numbers BK061675 and BK061828, and Supplementary data 1). Interestingly, the careful examination of the identified TLR15 gene sequences indicates that this gene is rendered non-functional in *H. raleighana* due to the occurrence of early sequence stop codons, while in *H. affinis* there is one functional allele and one rendered non-functional due to a frameshift mutation (**Supplementary Figures 2A, B**). The results in *H. affinis* were further confirmed by PCR amplification (**Supplementary Figure 2B**). Likewise, TLR15 orthologs were found in the two lungfish species (46, 47), which are representative of two extant families, Neoceratodontidae (*Neoceratodus forsteri*) (accession number BK061674) and Lepidosirenidae (*Protopterus annectens*). In NCBI, the Australian ghost shark *C. milii* TLR15 gene described here is incorrectly annotated as TLR6 (XM_042333155.1), while in the West African lungfish *P. annectens* the sequence presented here (Supplementary data 1) is an update (larger sequence encoding a signal peptide) to the incorrectly annotated TLR2 type-2 sequence (XM_044055095.1). The syntenic genomic block where TLR15 resides is conserved in vertebrates, although the flanking gene GPR75 is located elsewhere in all holocephalans analyzed (**Figure 1**). Additionally, a TLR15-like remnant was also found in tuatara in the conserved syntenic block. This remnant contains 1462 nucleotides spanning the final nine LRR described for chicken, the C terminal, TM domain, and TIR domain; however, it has several frameshift mutations and early stop codons (**Supplementary Data 1**).

TLRs are classified into seven families according to their ectodomain architecture and phylogenetic criteria (3, 8). In addition to the conserved synteny, our phylogenetic analyses support the identity of TLR15 in holocephalans and lungfish since the retrieved protein sequences cluster within the well-supported TLR15 clade both when using the full-length protein (**Figure 2**) and the ECD only (data not shown). Our phylogenetic analyses also show, with good support, that TLR15 forms a distinct subfamily within vertebrate TLRs and that it is most closely related to the TLR1 subfamily.

Putative TLR15 orthologs could not be found in mammals, amphibians, the coelacanth, ray-finned fish and elasmobranchs, despite the conserved synteny of the genes surrounding TLR15 (**Figure 1**). Likewise, our searches for putative TLR15 genes in jawless vertebrates did not retrieve any results. The searches were performed in several species of lamprey and in one hagfish (**Supplementary Table 1**), but only partial CDS of the syntenic genes could be located (**Figure 1**; **Supplementary Table 4**).

### Holocephalans and lungfish exhibit distinctive TLR15 protein structures

Motif prediction showed that the TLR15 in holocephalans and lungfish included the typical N-terminal signal peptide, ectodomain (ECD), transmembrane and TIR domains (**Supplementary Figure 6**).

**Figure 4:**
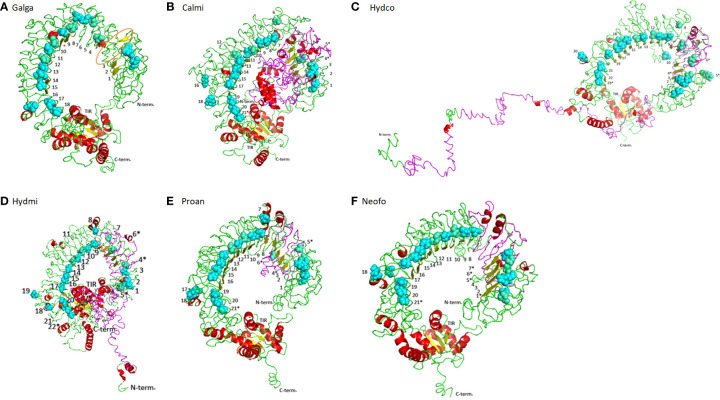
Structural conformation on cartoon mode of the TLR15 in different vertebrates. **(A)** Chicken TLR15 used as a model. The predicted long loop Chicken TLR15 used as a model. The predicted long loop between LRR3 and LRR4 modules is highlighted in orange and the LRR motif detected in other studies (48) is marked with a red asterisk; **(B)** *Callorhinchus milli*; **(C)** *Hydrolagus colliei*; **(D)** *Hydrolagus mirabilis*; **(E)** *Protopterus annectens*; and **(F)** *Neoceratodus forsteri*. The structures are shown in cartoon mode. The asparagines ladder positions are shown by sphere mode in cyan while the broken asparagines ladder are in greencyan. The LRR N-terminal (N-term) and C-terminal (C-term) and TIR domain detected modules are labeled and LRR modules are numbered.

In birds and reptiles, the TLR15 ECD is generally composed of a N-terminal LRR, a C-terminal LRR and 19 additional LRR motifs (48). Probably due to the different approaches used, in this study we were only able to detect 18 LRRs for the chicken TLR15 (**Supplementary Figures 1**, **Figure 4A**). Holocephalans and lungfishes present longer TLR15 proteins when compared to birds or reptiles (**Supplementary Figures 1, 6**). Indeed, the full TLR15 protein in these two lineages has upwards of 1007 aa while the chicken TLR15 has only 868 aa (**Supplementary Figure 1**). The major size differences are in the ectodomain region: taking the birds/reptiles TLR15 protein as reference, holocephalans exhibit 231 additional aa between the signal peptide and the first LRR motif described, and both holocephalans and lungfish have interval insertions of ~130 amino acids between the LRR3 and LRR4 (**Supplementary Figures 1, 4**). Structurally, the exclusive 231 aa region of holocephalan TLR15 modifies the protein conformation (**Figures 4B–D**) by disrupting its horseshoe structure. By using *H. affinis* cDNA, we were able to amplify part of these 231 aa insertions. Additionally, when we tested this larger insertion with different annotation tools (AUGUSTUS and GeneMarker) they did not infer any intron. In turn, the extra ~130 aa shared by holocephalans and lungfish does not seem to affect the typical horseshoe-shaped solenoid structure (**Figures 4B–F**). Interestingly, when compared to the chicken ortholog, holocephalan and lungfish TLR15 genes had five and four extra LRR motifs (marked with an asterisk in **Figures 4B–F**), respectively, with three of the extra LRRs being located in the inserted regions (**Supplementary Figure 1** and **Figure 4**). In contrast, the intracellular region of the protein (TIR domain) responsible for signal transduction is highly conserved across holocephalans, lungfish, birds and reptiles (**Supplementary Figures 1, 3**).

### TLR15 is weakly expressed in holocephalans

In this work, we used RNAseq data to analyze the TLR15 expression levels in different tissues of two holocephalans, *C. milii* and *H. colliei* (**Figure 3**). The patterns differed between species whereby TLR15 is expressed in all tissues of *C. milii*, with higher expression in gills, spleen, thymus and testis, while it is weakly expressed in *H. colliei* and only in heart, spleen, kidney and testis (**Figure 3**). When compared to two constitutively expressed TLR2 and TLR3 genes, TLR15 shows relatively low expression in both holocephalans, but more so in *H. colliei* (**Supplementary Figures 4, 5**).

### TLR15 is under negative selective pressure

Here, we searched for signatures of selection in the functional TLR15 proteins using the corresponding nucleotide sequences from three birds, three crocodilians, four squamate reptiles, two lungfishes and four holocephalans. Our results detected 7 sites under positive selection and 292 sites under negative selection (**Supplementary Tables 2**, **3**, respectively, and **Supplementary Figure 1**). From the 7 positively selected codons (PSC), six are located in the ectodomain. In turn, from de 292 sites under negative selection, 85 sites were detected in the TIR domain, 21 sites were in the C-terminus region and 75 sites were detected on the ECD, specifically in the LRR motifs. With the exception of LRR3 (no sites under selection), all LRR motifs are under negative pressure, with LRR18 exhibiting the highest number of sites under negative selection (7 sites).

## Discussion

Given their crucial role in the immune response against invading pathogens, TLRs are the most extensively studied PRRs. *TLR15* was first described in 2006 as being unique to birds (14), but later studies reported its occurrence in squamates (12) and cartilaginous fish (18). Comparative approaches suggested that these receptors originated before the divergence of cartilaginous fish and bony fish (~450mya) (49). Yet, the comparative genomics pipeline has been substantially modified in recent years, with additional genomes from representative lineages becoming available. In the present work, we make use of the newly available genomic and transcriptomic data (**Supplementary Table 1**) and expand the analysis of TLR15 origin and evolution to a wider array of vertebrate lineages, including lobe-finned fish, ray-finned fish, cartilaginous fish and jawless vertebrates (**Supplementary Table 1**). New TLR15 orthologs were identified in holocephalans and lungfish, and TLR15-like remnants was found in tuatara, based on amino acid sequence identity, protein structure, synteny analyses and phylogenetic reconstructions. This work expands the vertebrate groups previously reported as having the TLR15 gene and supports the idea transmitted in previous studies (6, 20) that TLR15 is an independent family more closely related to the TLR1 subfamily (**Figure 2**).

Syntenic blocks are considered important to identify orthologs since they provide an evolutionary informative genomic context (50). The chicken TLR15 is located downstream of the genes ankyrin repeat and SOCS box 3 (ASB3), Glutathione-specific gamma-glutamylcyclotransferase 2 (CHAC2) endoplasmic reticulum lectin 1 (ERLEC1), and upstream the genes probable G-protein coupled receptor 75 (GPR75), proteasome activator complex subunit 4 (PSME4) and Acylphosphatase 2 (ACYP2) (**Figure 1**). This genomic block is conserved in all vertebrate genomes we have investigated, including in holocephalans and lungfishes (**Figure 1**; **Supplementary Table 4**). Thus, our work supports earlier studies (20) proposing the presence of TLR15 in the ancestor of jawed vertebrates, and further shows that it has experienced multiple gene loss events along jawed vertebrate evolution, such as in elasmobranchs (sharks and rays), ray-finned fish (teleosts, holosteans, chondrosteans and polypterids), the coelacanth, amphibians, and mammals. Despite being present in Holocephalans, we did not detect a TLR15 ortholog in Elasmobranchs, the sister-lineage of Holocephalans that together comprise the Cartilaginous fish lineage. We searched the various genomes and transcriptomes currently available for the group [e.g (51)]. (**Supplementary Table 1**) and although the conserved synteny block was identified in all of them, we could not detect the TLR15 gene. Thus, it appears that TLR15 has been secondarily lost in sharks and rays. Additionally, similar gene losses and pseudogenization events were described in Sauropsids, including the archosaurs (birds and crocodilians), turtles, and lepidosaurs (tuatara and squamates) (6, 18, 19, 52, 53). In general, most species of archosaurs, turtles and lepidosaurs analyzed here showed TLR15 in genes in the conserved syntenic block but in all lineages, there were cases where no TLR15 was detected (e.g. *Eudyptes* penguins; in the softshell turtle *Pedoliscus sinensis;* and in the bearded dragon *Pogona vitticeps*) (18, 53) (**Supplementary Table 1**). Thus, evidence of secondary gene loss and pseudogenization appears widespread in sauropsids, including the basal lineage of tuatara (**Figure 1**).

In birds, squamates (14, 18), lungfishes and holocephalans, TLR15 is encoded by a single exon and is composed by a N-terminal signal peptide, an ectodomain, a transmembrane domain and the TIR domain (**Supplementary Figure 6**). Holocephalans present an extra 231 aa that disrupt the protein horseshoe-like structure, raising doubts on its true nature as part of a single exon. However, 1) successful amplification of this region was obtained in *H. affinis* using cDNA, suggesting it is indeed part of the TLR15 transcript; 2) the sequence translates into amino acids with no stop codons; 3) all TLR15 proteins have high similarity between the holocephalans studied; and 4) different annotation tools did not infer any putative intron; thus, the extra 231 aa in holocephalan TLR15 proteins appear to be indeed part of the coding region. The resulting impact of these extra 231aa to the protein function should be assessed in future functional studies. Furthermore, the extra ~130 aa found in holocephalans and lungfish TLR15 proteins is also present in the chicken TLR15 (**Figure 4A**), resulting in a long loop structure; however, no LRR motifs were detected there (48). The alignment of TLR15 sequences reveals an extensive diversification of the ectodomain (**Supplementary Figure 3**), suggesting that this region has been subject to different selective pressures likely due to the evolutionary arms race between pathogens and host. In turn, the TIR domain is the most conserved region in the protein (**Supplementary Figure 3**), being indicative of a slower evolutionary rate in contrast to the ectodomain, where the positions were considered as rapidly evolving.

TLRs originated more than 600 million years ago (54) and are able to detect a broad range of pathogens, being crucial for the host immune response. While the TLRs ectodomain seems to be evolving under positive selection in different lineages, the TIR domain remains highly conserved due to negative selection (44, 55–58). Here we show that TLR15 is under overall negative selection (**Supplementary Figure 1**; **Supplementary Table 3**), probably to maintain the protein conformation and biological role with a small proportion of codons under positive selection (**Figure 1** and **Supplementary Table 2**). Similar results have previously been published in the literature, where TLR15 in birds was described to be mainly under negative selection (48, 52). Here, the majority of the sites under negative selection are located in the TIR domain. Upon recognition of specific ligands by TLRs, the TIR domain initiates the downstream signal transduction, interacting with host adaptor proteins (59). It is known that the TIR domains of different TLRs are highly conserved across animals and plants, being under strong negative selection (60). Our results are in line with this expectation since 85 out of 144 amino acids that compose the TIR domain are under negative selection. Moreover, most of the residues described as being important for the TIR-TIR interface (48) are under negative forces in TLR15, as well as P1157 (**Supplementary Figure 1**) that is known to be essential for the MyD88-dependent signaling in mammalian TLRs (19, 61). Indeed, TLR15 signaling is presumed to occur *via* MyD88 with a downstream activation of nuclear factor -KB (NF-kB) that leads to the production of inflammatory cytokines, such as interleukin 1 beta (IL1β), IL6 and IL8 (19). In both holocephalans and lungfish, these adaptor and inflammatory proteins seem to be present in the genome and likely be functional (at least for *C. milii*; data not shown), supporting a functional TLR15 gene. In contrast, a more recent study describes that TLR15 in penguins is evolving under positive selection (53) with the majority of the sites under selection being located on the ectodomain. Our results also detected seven sites under positive selection, six of them being located in the ectodomain and one downstream of the predicted TIR domain. Since the TLRs ectodomain is the target for different pathogens, it was expected that the majority of the PSC would be located there. From these PSC, H325 (according to chicken sequence – **Supplementary Figure 1**) is located in a LRR motif and surrounded by N324 and I326 under negative selection, and P760 is located in the proline-rich loop described in chicken as important for protein cleavage (61), being also in close vicinity to P757 and R758 that are also under negative selection. As described above, these LRR motifs are important for pathogen detection (3, 62), thus variation in the nature of selection among specific lineages or even specific species may be modulated by differences in the pathogen communities and loads, leading to a specific optimization of the innate immune system.

TLRs, as other multigene families, often evolve by gene duplication, where the new gene copies may experience neofunctionalization, subfunctionalization or pseudogenization. Indeed, TLR15 shows clear signs of pseudogenization and gene loss in sauropsids (as discussed above). The bulk of the evidence gathered here further suggests that TLR15 is undergoing pseudogenization and/or possible loss of function in Holocephalans, notably (1): in some taxa, TLR15 is a pseudogene due to frameshift mutations and early stop codons (2); in taxa with continuous ORF for TLR15, the long insertion found in the proteins leads to the disruption of the characteristic horseshoe solenoid structure of the ECD, which is responsible for binding to PAMPs and thus essential to TLR function. Furthermore (3), TLR15 is weakly expressed in Holocephalans compared to other constitutively expressed TLR2 and TLR3 genes. Although we could detect only low expression of TLR15 in holocephalans (**Figure 3** and **Supplementary Figures 2**, **3**), it is known from the literature that it is highly expressed in different tissues and cells in birds, with higher expression in immune-relevant tissues such as the spleen and thymus (12, 63). In addition, there are no significant differences of expression between TLR2, TLR3 and TLR15 in birds, all of which are highly expressed (63), which is in great contrast to holocephalans. Such differences probably result from the adaptation to the environment and pathogen communities of each species or may also be due to a lower effector function of TLR15 in holocephalans.

## Conclusions

Overall, our comparative genomic approach provides a scenario for the evolution of TLR15 in vertebrates. We show for the first time that TLR15 is presented in cartilaginous fish (only in holocephalans) and in lungfish (i.e., basal lobe-fin fish), and tuatara (basal Lepidosauria) presented some TLR15-like remnants. Thus, we confirm that TLR15 was present in a jawed vertebrate ancestor, before the divergence of cartilaginous fish from other jawed vertebrates. Throughout vertebrate evolution, the TLR15 gene has been lost in multiple vertebrate lineages, namely ray-finned fish, the coelacanth, amphibians and mammals, and even within lineages (e.g., in Elasmobranchs). Although TLR15 is present in several species of holocephalans and in two species of lungfish, it is a pseudogene in some holocephalan taxa. In addition to holocephalans, there is evidence of ongoing gene loss in tuatara, turtles, and some penguins. To better understand the significance of these results further structural and functional studies should be performed.

## Data availability statement

The datasets presented in this study can be found in online repositories. The names of the repository/repositories and accession number(s) can be found in the article/**Supplementary Material**.

## Author contributions

FN and AV conceived the study. AM-M, AM and FN performed the bioinformatics searches. FN carried out the laboratory work, analyzed the data and drafted the manuscript. AV, LC, TA, AG and PE analyzed the data. AV analyzed the data and thoroughly edited the manuscript. All authors contributed to the article and approved the submitted version.
