# Corrigendum: Uncovering a 500 million year old history and evidence of pseudogenization for TLR15

**DOI:** 10.3389/fimmu.2023.1216338

**Published:** 2023-05-10

**Authors:** Fabiana Neves, Antonio Muñoz-Mérida, André M. Machado, Tereza Almeida, Arnaud Gaigher, Pedro J. Esteves, L. Filipe C. Castro, Ana Veríssimo

**Affiliations:** ^1^ CIBIO‐InBIO, Research Center in Biodiversity and Genetic Resources, University of Porto, Vairão, Portugal; ^2^ BIOPOLIS Program in Genomics, Biodiversity and Land Planning, CIBIO, Vairão, Portugal; ^3^ Department of Biology, Faculty of Sciences, University of Porto, Porto, Portugal; ^4^ CIIMAR - Interdisciplinary Centre of Marine and Environmental Research, University of Porto, Matosinhos, Portugal; ^5^ Research Group for Evolutionary Immunogenomics, Max Planck Institute for Evolutionary Biology, Plön, Germany; ^6^ Research Unit for Evolutionary Immunogenomics, Department of Biology, University of Hamburg, Hamburg, Germany; ^7^ CITS - Center of Investigation in Health Technologies, CESPU, Gandra, Portugal

**Keywords:** Toll-like receptor 15, evolution, pseudogenization, negative selection, jawed vertebrates

In the published article, there was an error in the **Funding** statement. One of the funding references was displayed as “EXPL/BIA-EVL/1562/2021” and should have been displayed as “EXPL/BIA-EVL/1045/2021”. The correct **Funding** statement appears below.

“This work was co-funded by the European Regional Development Fund (FEDER) and Norte Portugal Regional Operational Programme (NORTE2020), under the PORTUGAL 2020 Partnership Agreement, and by the project NORTE-01-0246-FEDER-000063. The authors also acknowledge research support *via* the projects PTDC/ASP-PES/28053/2017 – POCI-01-0145-FEDER-028053 co-funded by FEDER Funds through the Operational Competitiveness Factors Program COMPETE and by national funds through the Foundation for Science and Technology and EXPL/BIA-EVL/1045/2021, supported by the Foundation for Science and Technology. PE and AV, were supported by Portuguese funds through Portuguese Foundation for Science and Technology Contracts: CEECIND/CP1601/CT0005 (to PE) and DL57/2016 (to AV).”

The authors apologize for this error and state that this does not change the scientific conclusions of the article in any way. The original article has been updated.

