# Supplementary material for: Uncovering a 500 million year old history and evidence of pseudogenization for TLR15
Source: Front Immunol. 2022 Dec 20;13:1020601. doi: 10.3389/fimmu.2022.1020601 (PMC9808068; doi:10.3389/fimmu.2022.1020601)
Supplement: Supplementary file 1 [file DataSheet_1.zip › Data Sheet 1/Supplementary Figure 1.pdf]

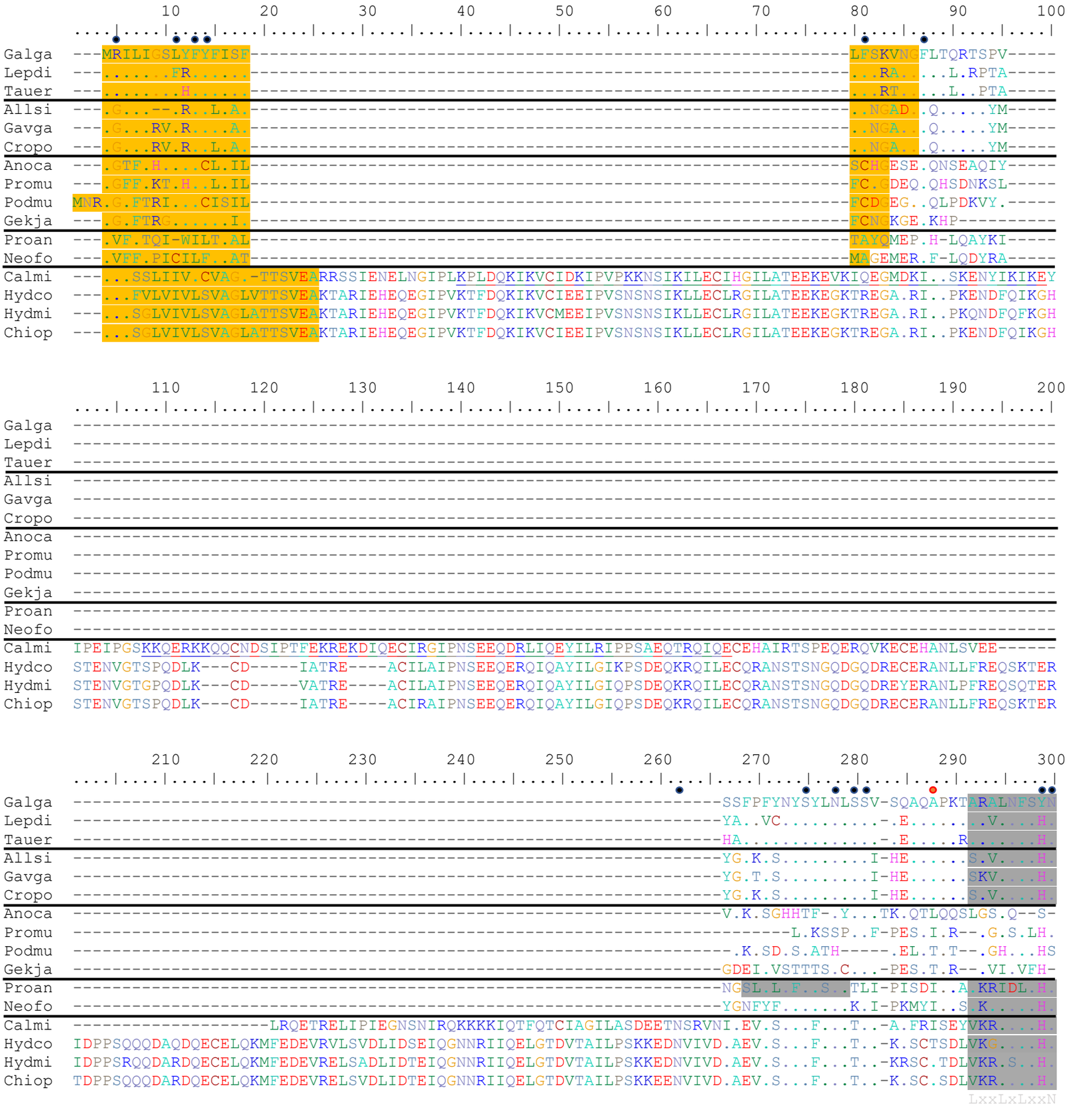
LxxLxLxxN

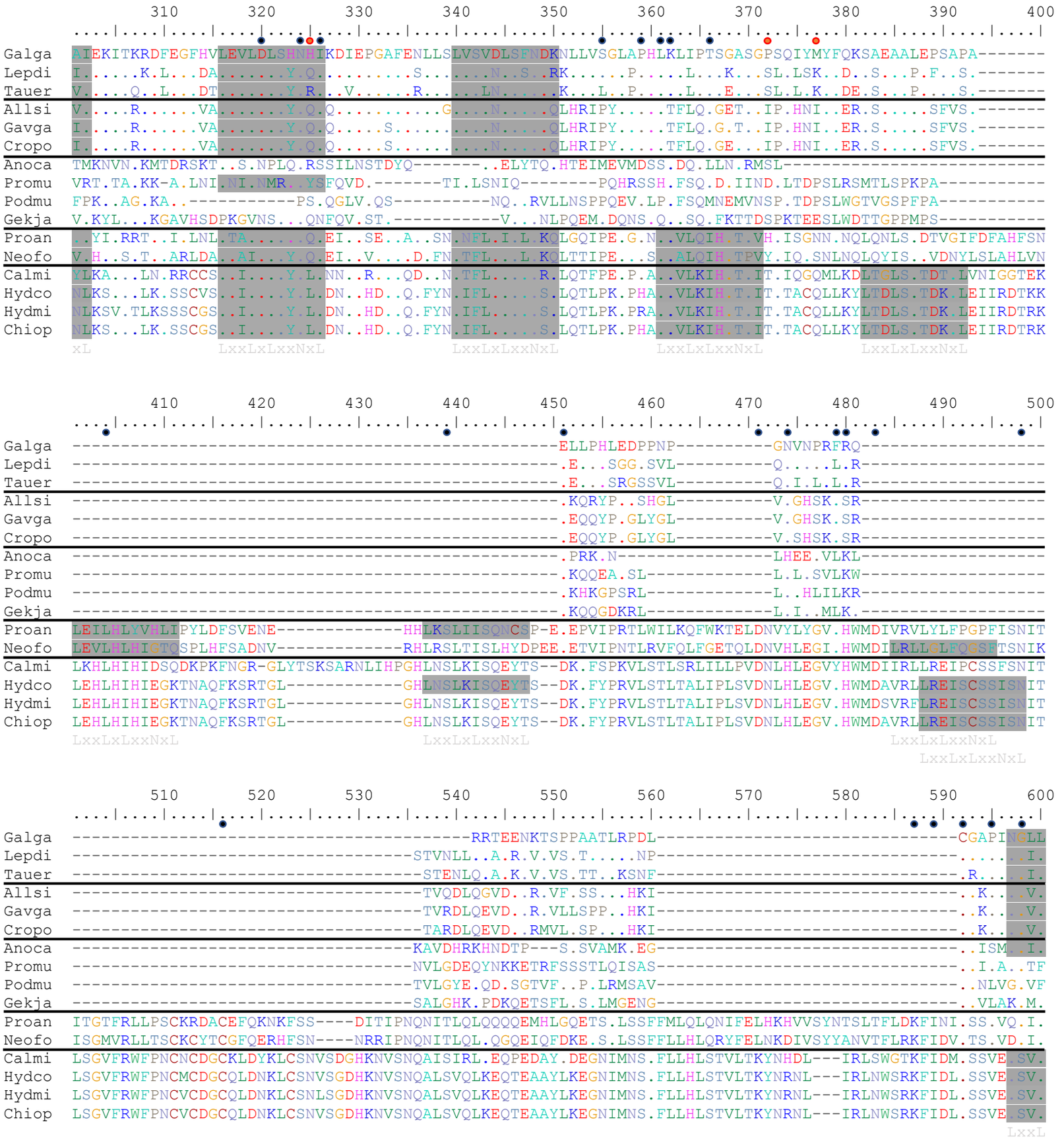
310 320 330 340 350 360 370 380 390 400
Galga AIEKITKRDFEGFHVLEVLDLSHNHIKDIEPGAFENLLSLVSVDLSFNDKNLLVSGLAPHLKLIPTSGASGPSQIYMYFQKSAEAALEPSAPA-------
Lepdi I.....K.L...DA.....Y.Q.........S.....N..S..RK......P.....L...K...SL.LSK..D..S...P.F..S.-------
Tauer V.....Q..L...DT.....Y.R...V......R.....LN.....K...L..P.....L...E...SL.L.K..DE.S...P....S.-------
Allsi V.....R......VA.....Y.Q.Q..........G....N.....QLHRIPY.....TFLQ.GET..IP.HNI..ER.S.....SFVS.-------
Gavga I.....R......VA.....Y.Q.Q.....S.....G....N.....QLHRIPY.....TFLQ.G.T..IP.HNI..ER.S.....SFVS.-------
Cropo I.....R......VA.....Y.Q.Q.....S.....G....N.....QLHRIPY.....TFLQ.GE...IP.HNI..ER.S.....SFVS.-------
Anoca TMKNVN.KMTDRSKT..S.NPLQ.RSSILNSTDYQ-------..ELYTQ.HTEIMEVMDSS.DQ.LLN.RMSL------------------------
Promu VRT.TA.KK-A.LNI.NI.NMR..YSFQVD.-------TI.LSNIQ--------PQHRSSH.FSQ.D.IIND.LTDPSLRSMTLSPKPA----------
Podmu FPK..AG.KA..----------PS.QGLV.QS----------NQ..RVLLNSPPQEV.LP.FSQMNEMVNSP.TDPSLWGTVGSPFPA----------
Gekja V.KYL...KGAVHSDPKGVNS...QNFQV.ST.----------V...NLPQEM.DQNS.Q..SQ.FKTTDSPKTEESLWDTTGPPMPS----------
Proan ..YI.RRT..I.LNL.TA.....Q.EI..SE..A..SN.NFL.I.L.KQLGQIPE.G.N..VLQIH.T.VH.ISGNN.NQLQNLS.DTVGIFDFAHFSN
Neofo V.H..S.T..ARLDA..AI...Y.Q.EI..V....D.FN.TFL...L.KQLTTIPE...S..ALQIH.TPVY.IQ.SNLNQLQYIS..VDNYLSLAHLVN
Calmi YLKA...LN.RRCCS..I...Y.L.NN..R...QD..N.TFL.....R.LQTFPE.P.A..VLKIH.T.IT.IQGQMLKDLTGLS.TDT.LVNIGGTEK
Hydco NLKS...LK.SSCVS..I...Y.L.DN..HD..Q.FYN.IFL.....S.LQTLPK.PHA..VLKIH.T.IT.TACQLLKYLTDLS.TDK.LEIIRDTKK
Hydmi NLKSV.TLKSSSCGS..I...Y.L.DN..HD..Q.FYN.IFL.....S.LQTLPK.PRA..VLKIH.T.IT.TACQLLKYLTDLS.TDK.LEIIRDTRK
Chiop NLKS...LK.SSCGS..I...Y.L.DN..HD..Q.FYN.IFL.....S.LQTLPK.PHA..VLKIH.T.IT.TACQLLKYLTDLS.TDK.LEIIRDTRK
xL LxxLxLxxNxL LxxLxLxxNxL LxxLxLxxNxL LxxLxLxxNxL
410 420 430 440 450 460 470 480 490 500
Galga ----------ELLPHLEDPPNP----------GNVNPRFRQ----------
Lepdi ----------.E...SGG.SVL----------Q.....L.R----------
Tauer ----------.E...SRGSSVL----------Q.I.L.L.R----------
Allsi ----------.KQRYP..SHGL----------V.GHSK.SR----------
Gavga ----------.EQQYP.GLYGL----------V.GHSK.SR----------
Cropo ----------.EQQYP.GLYGL----------V.SHSK.SR----------
Anoca ----------.PRK.N----------LHEE.VLKL----------
Promu ----------.KQQEA.SL----------L.L.SVLKW----------
Podmu ----------.KHKGPSRL----------L..HLILKR----------
Gekja ----------.KQQGDKRL----------L.I..MLK.----------
Proan LEILHLYVHLIPYLDFSVENE----------HHLKSLIISQNCSP-E.EPVIPRTLWILKQFWKTELDNVYLYGV.HWMDIVRVLYLFPGPFISNIT
Neofo LEVLHLHIGTQSPLHFSADNV----------RHLRSLTISLHYDPEE.ETVIPNTLRVFQLFGETQLDNVHLEGI.HWMDILRLLGLFQGSFTSNIK
Calmi LKHLHIHIDSQDKPKFNGR-GLYTSKSARNLIHPGHLNSLKISQEYTS--DK.FSPKVLSTLSRLILLPVDNLHLEGVYHWMDIIRLLREIPCSSFSNIT
Hydco LEHLHIHIEGKTNAQFKSRTGL----------GHLNSLKISQEYTS--DK.FYPRVLSTLTALIPLSVDNLHLEGV.HWMDAVRLLREISCSSISNIT
Hydmi LEHLHIHIEGKTNAQFKSRTGL----------GHLNSLKISQEYTS--DK.FYPRVLSTLTALIPLSVDNLHLEGV.HWMDSVRFLREISCSSISNIT
Chiop LEHLHIHIEGKTNAQFKSRTGL----------GHLNSLKISQEYTS--DK.FYPRVLSTLTALIPLSVDNLHLEGV.HWMDAVRLLREISCSSISNIT
LxxLxLxxNxL LxxLxLxxNxL LxxLxLxxNxL LxxLxLxxNxL
510 520 530 540 550 560 570 580 590 600
Galga ----------RRTEENKTSPPAATLRPDL----------CGAPINGLL
Lepdi ----------STVNLL..A.R.V.VS.T.....NP----------.....I.
Tauer ----------STENLQ.A.K.V.VS.TT..KSNF----------.R....I.
Allsi ----------TVQDLQGVD..R.VF.SS...HKI----------..K...V.
Gavga ----------TVRDLQEVD..R.VLLSPP..HKI----------..K...V.
Cropo ----------TARDLQEVD..RMVL.SP...HKI----------..K...V.
Anoca ----------KAVDHRKHNDTP---S.SVAMK.EG----------..ISM..I.
Promu ----------NVLGDEQYNKKETRFSSSTLQISAS----------..I.A..TF
Podmu ----------TVLGYE.QD.SGTVF..P.LRMSAV----------..NLVG.VF
Gekja ----------SALGHK.PDKQETSFL.S.LMGENG----------..VLAK.M.
Proan ITGTFRLLPSCKRDACEFQKNKFSS----DITIPNQNITLQLQQQQEMHLGQETS.LSSFFMLQLQNIFELHKHVVSYNTSLTFLDKFINI.SS.VQ.I.
Neofo ISGMVRLLTSCKCYTCGFQERHFSN----NRRIPNQNITLQL.QGQEIQFDKE.S.LSSFFLLHLQRYFELNKDIVSYYANVTFLRKFIDV.TS.VD.I.
Calmi LSGVFRWFPNCNCDGCKLDYKLCSNVSDGHKNVSNQAISIRL.EQPEDAY.DEGNIMNS.FLLHLSTVLTKYNHDL---IRLSWGTKFIDM.SSVE.SV.
Hydco LSGVFRWFPNCMCDGCQLDNKLCSNVSGDHKNVSNQALSVQLKEQTEAAYLKEGNIMNS.FLLHLSTVLTKYNRNL---IRLNWSRKFIDL.SSVE.SV.
Hydmi LSGVFRWFPNCVCDGCQLDNKLCSNLSGDHKNVSNQALSVQLKEQTEAAYLKEGNIMNS.FLLHLSTVLTKYNRNL---IRLNWSRKFIDL.SSVE.SV.
Chiop LSGVFRWFPNCVCDGCQLDNKLCSNVSGDHKNVSNQALSVQLKEQTEAAYLKEGNIMNS.FLLHLSTVLTKYNRNL---IRLNWSRKFIDL.SSVE.SV.
LxxL

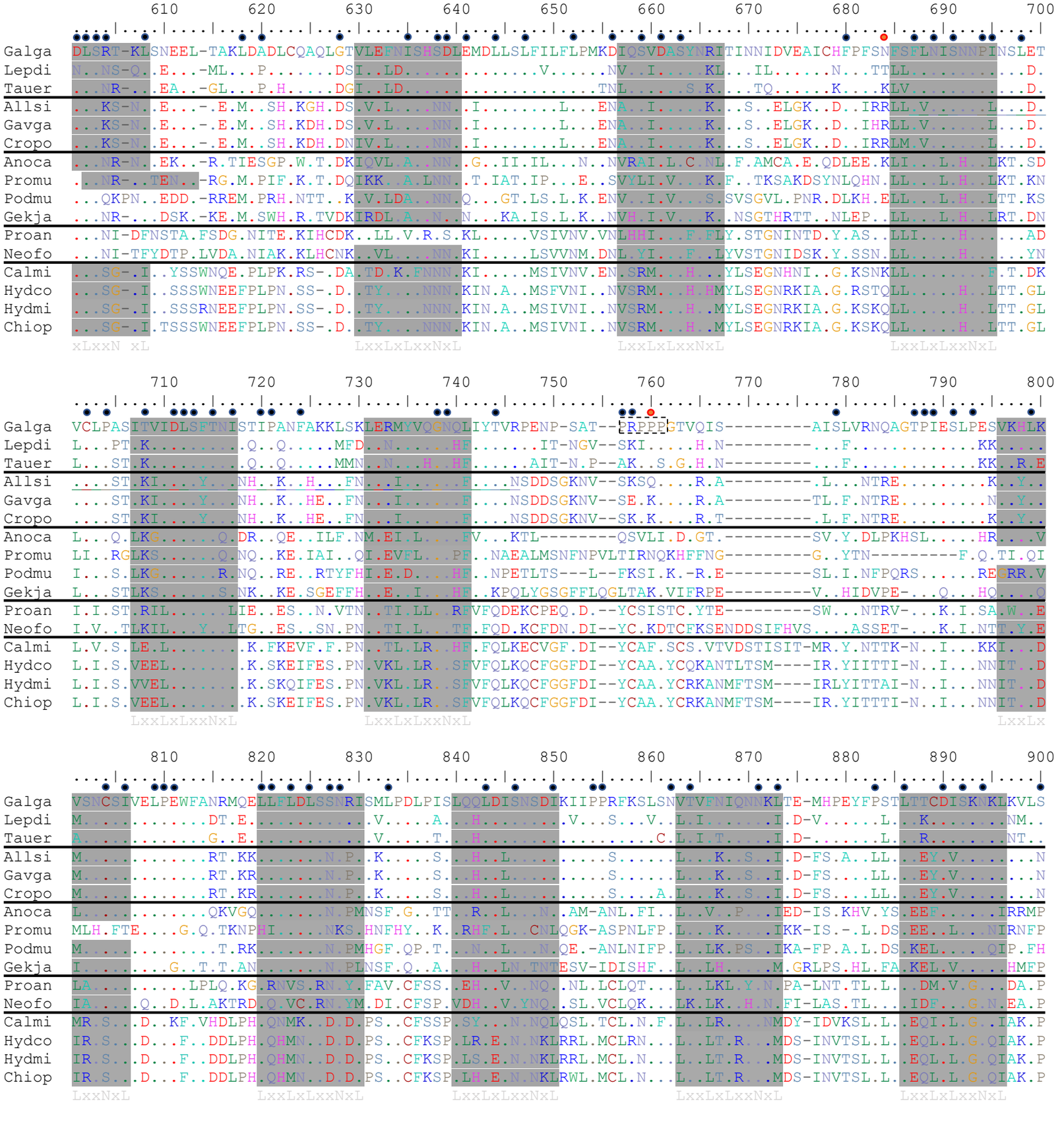
610 620 630 640 650 660 670 680 690 700
Galga DLSRT-KLSNEEL-TAKLDADLCQAQLGTVLEFNISHSDLEMDLLSLFILFLPMKDIQSVDASYNRITINNIDVEAICHFPFSNFSFLNISNNPINSLET
Lepdi N..NS-Q..E...-ML...P.......DSI..LD.....V.....NV..I.....KL...IL.....N...TTLL.....D.
Tauer ...NR-...EA..-GL...P.H.....DGI..LD.....TNL.....S.K...TQ.....K....KLV.....D.
Allsi ...KS-N..E...-.E.M..SH.KGH.DS.V.L...NN..I.....L...ENA..I.....K...S..ELGK..D..IRRLL.V.....L...D.
Gavga ...KS-N..E...-.E.M..SH.KDH.DS.V.L...NN..I.....L...ENA..I.....K...S..ELGK..D..IHRLL.V.....L...D.
Cropo ...KS-N..E...-.E.M..SH.KDH.DNIV.L...NN..I.....L...ENA..I.....K...S..ELGK..D..IRRLM.V.....L...D.
Anoca ...NR-N..EK..-R.TIESGP.W.T.DKIQVL.A..NN..G..II.IL...N..NVRAI.L.C.NL.F.AMCA.E.QDLEE.KLI...L.H..LKT.SD
Promu ...NR-..TEN..-RG.M.PIF.K.T.DQIKK..A.LNN..T.IAT.IP...E..SVYLI.V...K.F..TKSAKDSYNLQHN.LL...L.H..LKT.KN
Podmu ...QKPN..EDD.-RREM.PRH.NTT..K.V.LDA..NN.Q...GT.LS.L.K.ENV..I.V...S.SVSGVL.PNR.DLKH.ELL...L.H..LTT.KS
Gekja ...NR-...DSK.-KE.M.SWH.R.TVDKIRDL.A..N..N...KA.IS.L.K..NVH.I.V...K..NSGTHRTT..NLEP..LL...L.H..LRT.DN
Proan ...NI-DFNSTA.FSDG.NITE.KIHCDK..LL.V.R.S.KL.....VSIVNV.VNLHHI...F.FLY.STGNINTD.Y.AS..LLI....H.....AD
Neofo ...NI-TFYDTP.LVDA.NIAK.KLHCNK..VL....NN.KI.....LSVVNM.DNL.YI...F..LYVSTGNIDSK.Y.SSN.LL....H.....YN
Calmi ...SG-.I..YSSWNQE.PLPK.RS-.DA.TD.K.FNNN.KI.....MSIVNV.EN.SRM...H...YLSEGNHNI..G.KSNKLL.....F.T.DK
Hydco ...SG-.I..SSSWNEEFPLPN.SS-.D..TY....NNN.KIN.A..MSFVNI..NVSRM...H.HMYLSEGNRKIA.G.RSTQLL.....H..LTT.GL
Hydmi ...SG-.I..SSSRNEEFPLPN.SS-.D..TY....NNN.KIN.A..MSIVNI..NVSRM...H..MYLSEGNRKIA.G.KSKQLL.....H..LTT.GL
Chiop ...SG-.I.TSSSWNEEFPLPN.SS-.D..TY....NNN.KIN.A..MSIVNI..NVSRM...H..MYLSEGNRKIA.G.KSKQLL.....H..LTT.GL
xLxxN xL LxxLxLxxNxL LxxLxLxxNxL LxxLxLxxNxL
710 720 730 740 750 760 770 780 790 800
Galga VCLPASITVIDLSFTNISTIPANFAKKLSKLERMYVQGNQLIYTVRPENP-SAT--PRPPPSTVQIS---------AISLVRNQAGTPIESLPESVKHLK
Lepdi L...PT.K.....Q..Q.....MFD..N.....HF.....IT-NGV--SKI.....H.N---------...F.....KK.....
Tauer L...ST.K.....Q..Q.....MMN..N...H..HF.....AIT-N.P--AK..S.G.H.N---------...F.....KK..R.E
Allsi ....ST.KI...Y...NH..K..H...FN...I.....F....NSDDSGKNV--SKSQ....R.A---------.L...NTRE.....K..Y..
Gavga ....ST.KI...Y...NH..K..HE..FN...I.....F....NSDDSGKNV--SE.K....R.A---------TL.F.NTRE.....N..Y..
Cropo ....ST.KI...Y...NH..K..HE..FN...I.....F....NSDDSGKNV--SK.K....R.T---------.L.F.NTRE.....K..Y..
Anoca L...Q.LKG.....Q.DR..QE..ILF.NM.EI.L....FV...KTL--------QSVLI.D.GT.---------SV.Y.DLPKHSL.....HR....V
Promu LI..RGLKS.....Q.NQ..KE.IAI..QI.EVFL...PF..NAEALMSNFNPVLTIRNQKHFFNG---------G..YTN---------F.Q.TI.QI
Podmu I...S.LKG.....R.NQ..RE..RTYFHI.E.D....HF..NPETLTS---L--FKSI.K.-R.E---------SL.I.NFPQRS.....REGRR.V
Gekja L...STLKS.....S.NK..KE.SGEFFH..E..I...HF..KPQLYGSGFFLQGLTAK.VIFRPE---------V..HIDVPE-...Q....HQ....Q
Proan I.I.ST.RIL.....LIE..ES..N.VTN..TI.LL..RFVFQDEKCPEQ.D.--YCSISTC.YTE---------SW...NTRV-...K.I.SA.W..E
Neofo I.V..TLKIL...Y..LTG..ES..SN.PN..TI.L...TF.FQD.KCFDN.DI--YC.KDTCFKSENDDSIFHVS....ASSET-...K.I.NTT.Y.E
Calmi L.V.S.LE.L.....K.FKEVF.F.PN..TL.LR..HF.FQLKECVGF.DI--YCAF.SCS.VTVDSTISIT-MR.Y.NTTK-N..I...KKI...D
Hydco L.I.S.VEEL.....K.SKEIFES.PN.VKL.LR..SFVFQLKQCFGGFDI--YCAA.YCQKANTLTSM----IR.YIITTI-N..I...NNIT..D
Hydmi L.I.S.VVEL.....K.SKQIFES.PN.VKL.LR..SFVFQLKQCFGGFDI--YCAA.YCRKANMFTSM----IRLYITTAI-N..I...NNIT..D
Chiop L.I.S.VEEL.....K.SKEIFES.PN.VKL.LR..SFVFQLKQCFGGFDI--YCAA.YCRKANMFTSM----IR.YITTTI-N..I...NNIT..D
LxxLxLxxNxL LxxLxLxxNxL LxxLx
810 820 830 840 850 860 870 880 890 900
Galga VSNCSIVELPEWFANRMQELLFLDLSSNRISMLPDLPISLQQLDISNSDIKIIPPRFKSLSNVTVFNIQNNKLTE-MHPEYFPSTLTTCDISKNKLKVLS
Lepdi M.....DT.E.....V.....A..H.....V...S...V..L.I.....I.D-V.....L..K.....NM..
Tauer A.....G..E.....V.....T..H.....C.L.I.T.....I.D-.....L..R.....NT..
Allsi M.....RT.KK.....N.P..K.....S..H..L.....S.....L...K..S..I.D-FS.A..LL..EY.V.....N
Gavga M.....RT.KR.....N.P..K.....S..H..L.....S.....L...K..S..I.D-FS....LL..EY.V.....N
Cropo M.....RT.KR.....N.P..K.....S..H..L.....S....A.L...K..S..I.D-FS....LL..EY.V.....N
Anoca L.....QKVGQ.....N.PMNSF.G..TT..R..L...N..AM-ANL.FI..L..V..P...IED-IS.KHV.YS.EEF.....IRRMP
Promu MLH.FTE....G.Q.TKNPHI.....NKS.HNFHY..K..RHF.L..CNLQGK-ASPNLFP.L...K.....IKK-IS.-.L.DS.EE..L...NIRNFP
Podmu M.....T.RK.....N.PMHGF.QP.T...N..L...N.QE.-ANLNIFP.L..LK.PS..IKA-FP.A.L.DS.KEL.....QIP.FH
Gekja I.....G..T.T.AN.....N.PLNSF.Q..A...H..LN.TNTESV-IDISHF..L..LH.....M.GRLPS.HL.FA.KEL.V.....HMFP
Proan LA.....LPLQ.KG.RNVS.RN.Y.FAV.CFSS..EH..V..NQ..NL.LCLQT...L..LKL.Y.N.PA-LNT.TL.L...DM.V.G...DA.P
Neofo IA.....Q..D.L.AKTRD.Q.VC.RN.YM.DI.CFSP.VDH..V.YNQ..SL.VCLQK...LK.LK..H.N.FI-LAS.TL....IDF...G.N.EA.P
Calmi MR.S...D..KF.VHDLPH.QNMK..D.D.PS..CFSSP.SY...N.NQLQSL.TCL.N.F.L..LR.....NMDY-IDVKSL.L..EQI.L.G..IAK.P
Hydco IR.S...D...F..DDLPH.QHMN..D.D.PS..CFKSP.LR.E.N.NKLRRL.MCLRN...L..LT.R...MDS-INVTSL.L..EQL.L.G.QIAK.P
Hydmi IR.S...D...F..DDLPH.QHMN..D.D.PS..CFKSP.LS.E.N.NKLRRL.MCL.N...L..LT.R...MDS-INVTSL.L..EQL.L.G.QIAK.P
Chiop IR.S...D...F..DDLPH.QHMN..D.D.PS..CFKSP.LH.E.N.NKLRWL.MCL.N...L..LT.R...MDS-INVTSL.L..EQL.L.G.QIAK.P
LxxNxL LxxLxLxxNxL LxxLxLxxNxL LxxLxLxxNxL LxxLxLxxNxL

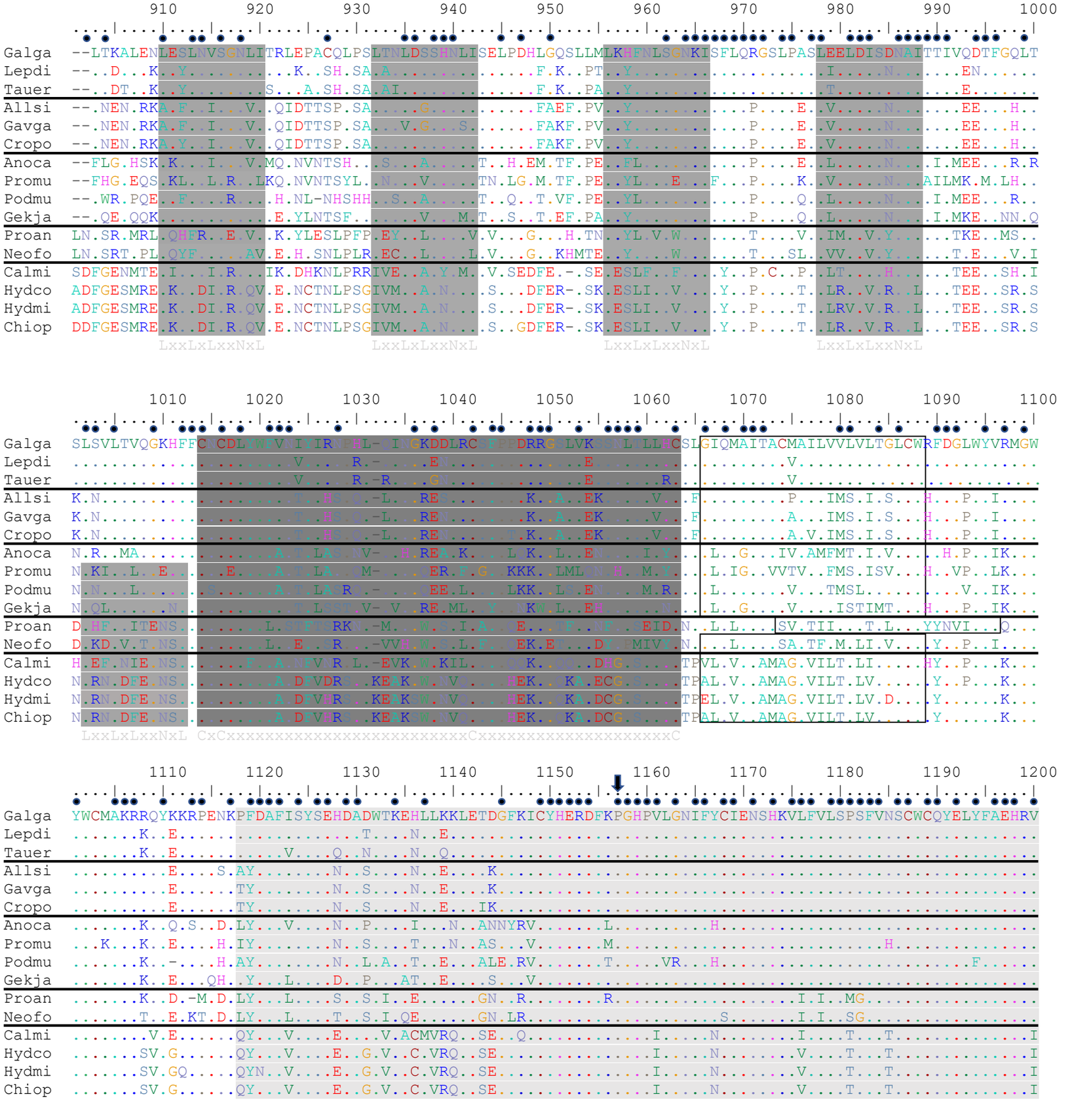
910 920 930 940 950 960 970 980 990 1000
Galga --LTKALENLESLNVSGNLITRLEPACQLPSLTNLDSSHNLISELPDHLGQSLLMLKHFNLSGNKISFLQRGSLPASLEELDISDNAITTIVQDTFGQLT
Lepdi --..D...K..Y.....K..SH.SA.A.....F.K..PT..Y.....I.....N.....EN.....
Tauer --..DT..K..Y.....S...A.SH.SA.AI.....F.K..PA..Y.....T.....E.....
Allsi --.NEN.RKA.F..I...V..QIDTTSP.SA.....G.....FAEF.PV..Y.....P....E..V.....N.....EE...H..
Gavga --.NEN.RKA.F..I...V..QIDTTSP.SA...V.G...S.....FAKF.PV..Y.....P....E..V.....N.....EE...H..
Cropo --.NEN.RKA.Y..I...V..QIDTTSP.SA.....FAKF.PV..Y.....P....E..V.....N.....EE...H..
Anoca --FLG.HSK.K...I...V.MQ.NVNTSH...S...A.....T..H.EM.TF.PE..FL.....P....E..L.....N....I.MEE...R.R
Promu --FHG.EQS.KL..L.R..LKQ.NVNTSYL..N...V.....TN.LG.M.TF.PE..YL...E...F...P....K..V.....N...AILMK.M.LH..
Podmu --.WR.PQE..F....R...H.NL-NHSHH..S..A.....T..Q..T.VF.PE..YL.....P....Q..L.....N....I.MEE...R..
Gekja --.QE.QQK.....E.YLNTSF.....V...M.T..S..T.EF.PA..Y.....P....Q..L.....N....I.MKE..NN.Q
Proan LN.SR.MRL.QHFR..E.V..K.YLESLPFP.EY..L...V.V...G...H.TN..YL.V.W.....T....V..IM..V.Y.....TKE..MS..
Neofo LN.SRT.PL.QYF....AV.E.H.SNLPLR.EC..L...L.V...G..KHMTE..Y...W.....T...SL..VV..V.Y.....T.E...V.I
Calmi SDFGENMTE.I...I.R...IK.DHKNLPRRIVE..A.Y.M..V.SEDFE.-.SE.ESLF..F....Y..P.C..P..LT....H.....TEE..SH.I
Hydco ADFGESMRE.K..DI.R.QV.E.NCTNLPSGIVM..A.N...S...DFER-.SK.ESLI..V....Y..P....T..LR..V.R..L...TEE..SR.S
Hydmi ADFGESMRE.K..DI.R.QV.E.NCTNLPSGIVM..A.N...S...DFER-.SK.ESLI..V....Y..P....T..LRV.V.R..L...TEE..SR.S
Chiop DDFGESMRE.K..DI.R.QV.E.NCTNLPSGIVM..A.N...S..GDFER-.SK.ESLI..V....Y..P....T..LR..V.R..L...TEE..SR.S
LxxLxLxxNxL LxxLxLxxNxL LxxLxLxxNxL LxxLxLxxNxL
1010 1020 1030 1040 1050 1060 1070 1080 1090 1100
Galga SLSVLTVQGKHFFCDCDLYWFVNIYIRNPPL-QINGKDDLRCSPPDRRGLVKSSNLTLLHCSLGIQMAITACMAILVVLVLTGLCWRFDGLWYVRMGW
Lepdi ..........V...R.-...EN.....E.....V.....
Tauer ..........V...R.-R...GN.....E....R.....V.....
Allsi K.N.....T..HS.Q.-L..RES.....K..A..EK...V..F.....P...IMS.I.S...H...P..I....
Gavga K.N.....T..HS.Q.-L..REN.....K..A..EK...V..F.....A...IMS.I.S...H...P..I....
Cropo K.N.....T..HS.Q.-L..REN.....T.K..A..EK...V..F.....A.V.IMS.I.S...H...P..I....
Anoca N.R..MA.....A.T.LAS.NV-..H.REA.K...L.K..L..EN...I.Y...L..G...IV.AMFMT.I.V....H.P..IK...
Promu N.KI..L..E....E....A.T.LA...QM-...ER.F.G.KKK..LML.N.H..M.Y...L.IG..VVTV..FMS.ISV...H..VP..LK...
Podmu N.N...L.....S.....A.T.LAS.R.-...EE.L...LKK..L.S.EN...M.R...L.....V...TMSL.....V..IK...
Gekja N.QL.....N.....T.LSST.V-.V..RE.ML..Y..NKW.L..EH.....N...L..G....V....ISTIMT...H...P..IK...
Proan D.HF..ITENS.....L.STPTSRKN.-M...W.S.I.A..QE...TF..NF..SEID.N..L.L....SV.TII...T.L...YYNVI...Q...
Neofo D.KD.V.T.NS.....L.E...SR...-VVH.W.S.L.E..QEK.E.T...DY.PMIVY.N....L....SA.TF.M.LI.V....Y..P..I....
Calmi H.EF.NIE.NS.....F..A.NEVNR.L.-EVK.W.KIL...QCK..QG..DHG.S....TPVL.V..AMAG.VILT.LI.....HY..P...K...
Hydco N.RN.DFE.NS.....A.DEVDR...KEAK.W.NVQ...HEK...KA.ECG.S....TPAL.V..AMAG.VILT.LV.....Y..P...K...
Hydmi N.RN.DFE.NS.....A.DEVHR...KEAK.W.NVQ...HEK...KA.DCG.S....TPEL.V..AMAG.VILT.LV.D...Y.....K...
Chiop N.RN.DFE.NS.....A.DEVHR...KEAK.W.NVQ...HEK...KA.DCG.S....TPAL.V..AMAG.VILT.LV.....Y.....K...
LxxLxLxxNxL CxCxxxxxxxxxxxxxxxxxxxxxxxxxxCxxxxxxxxxxxxxxxxxxxC
1110 1120 1130 1140 1150 1160 1170 1180 1190 1200
Galga YWCMAKRRQYKKRPENKPFDAFISYSEHDADWTKEHLLKKLETDGFKICYHERDFKPGHPVLGNIFYCIENSHKVLFVLSPSFVNSCWCQYELYFAEHRV
Lepdi .....K..E.....T....N..E.....
Tauer .....K..E.....V....Q..N....N..Q.....
Allsi .....E....S.AY.....N..S....N..E....K.....
Gavga .....E.....TY.....N..S....N..E....K.....
Cropo .....E.....TY.....N..S....N..E...IK.....
Anoca .....K..Q.S..D.LY...V....N..P....I...N..ANNYRV.....L.....H.....
Promu ...K...K..E....H.IY.....N..S....T...N..AS...V.....M.....H.....
Podmu .....K..-....H.AY.....N..L.A..T..E...ALE.RV.....T.....VR...H.....F.....
Gekja .....K..E...QH..Y...L....D..P...AT..E....S...V.....
Proan .....K..D.-M.D.LY...L....S..S.I..E.....GN..R.....R.....I.I..MG.....
Neofo .....T..E.KT.D.LY...L....T..S.I.QE.....GN.LR.....S.....I.I..SG.....
Calmi .....V.E.....QY...V....E....V.ACMVRQ..SE..Q.....I.....N.....I....T...T.....I
Hydco .....SV.G.....QY...V....E..G.V..C.VRQ..SE.....I.....N.....V....T...T.....I
Hydmi .....SV.GQ.....QYN..V....E..G.V..C.VRQ..SE.....I.....N.....V....T...T.....I
Chiop .....SV.G.....QY...V....E..G.V..C.VRQ..SE.....I.....N.....V....T...T.....I

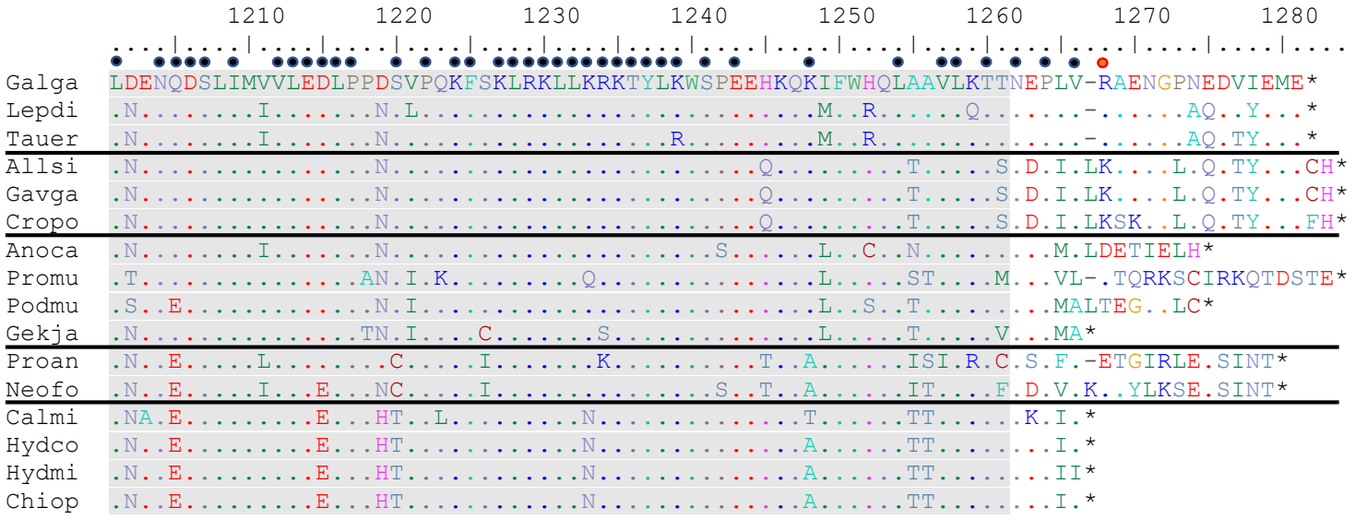
1210
1220
1230
1240
1250
1260
1270
1280
Galga LDENQDSLIMVVLEDLPPDSVPQKFSKLRKLLKRKTYLKWSPEEHKQKIFWHQLAAVLKTTNEPLV-RAENGPNEDVIEME*
Lepdi .N.....I.....N.L.....M..R.....Q.....-.....AQ..Y...*
Tauer .N.....I.....N.....R.....M..R.....-.....AQ.TY...*
Allsi .N.....N.....Q.....T.....S.D.I.LK....L.Q.TY...CH*
Gavga .N.....N.....Q.....T.....S.D.I.LK....L.Q.TY...CH*
Cropo .N.....N.....Q.....T.....S.D.I.LKSK..L.Q.TY...FH*
Anoca .N.....I.....N.....S.....L..C..N.....M.LDETIELH*
Promu .T.....AN.I.K.....Q.....L.....ST....M...VL-.TQRKSCIRKQTDSTE*
Podmu .S..E.....N.I.....L..S..T.....MALTEG..LC*
Gekja .N.....TN.I....C.....S.....L.....T.....V...MA*
Proan .N..E.....L.....C.....I.....K.....T..A.....ISI.R.C.S.F.-ETGIRLE.SINT*
Neofo .N..E.....I...E...NC.....I.....S..T..A.....IT....F.D.V.K..YLKSE.SINT*
Calmi .NA.E.....E...HT..L.....N.....T.....TT.....K.I.*
Hydco .N..E.....E...HT.....N.....A.....TT.....I.*
Hydmi .N..E.....E...HT.....N.....A.....TT.....II*
Chiop .N..E.....E...HT.....N.....A.....TT.....I.*
