# Supplementary material for: Uncovering a 500 million year old history and evidence of pseudogenization for TLR15
Source: Front Immunol. 2022 Dec 20;13:1020601. doi: 10.3389/fimmu.2022.1020601 (PMC9808068; doi:10.3389/fimmu.2022.1020601)
Supplement: Supplementary file 1 [file DataSheet_1.zip › Data Sheet 1/Supplementary Figure 3.pdf]

# ConSurf Results

```
1
M R I L I G S L Y F
e e b b b b b b b b
f

11
Y F I S F L F S K V
b b b b b b b e e e

21
N G F L T Q R T S P
e e b e e e e e e e

31
V S S F P F Y N Y S
b e b e e b b e b b
              f

41
Y L N L S S V S Q A
b b e b e e b e e e
    f   f f

51
Q A P K T A R A L N
e b e e e b e e b e

61
F S Y N A I E K I T
b b e e b b e e b b
    f f

71
K R D F E G F H V L
e e e b e e b e b b

81
E V L D L S H N H I
e b b e b b e e e b
      f       f

91
K D I E P G A F E N
e e b e e e b b e e

101
L L S L V S V D L S
b b e b b b b e b e
                  f

111
F N D K N L L V S G
b e e e e e e b e e
  f

121
L A P H L K L I P T
b e e e b e b b e e
          f

131
S G A S G P S Q I Y
e e e e e e e e b e

141
M Y F Q K S A E A A
e b b e e b e e e e
                  f

151
L E P S A P A E L L
b e e e e e e e e e
              f

161
P H L E D P P N P G
e e e e e b e b b b

171
N V N P R F R Q R R
e b e e e b e e e e

181
T E E N K T S P P A
e e e e e e e e e e

191
A T L R P D L C G A
b e b e e e b b e e
              s

201
P I N G L L D L S R
e e e b b b e b b e
            f s s

211
T K L S N E E L T A
e e b e e e e b e e

221
K L D A D L C Q A Q
e b e e e e b e e e
            s

231
L G T V L E F N I S
b e e b b e b b b b
                  s

241
H S D L E M D L L S
e e e b e b e b b b
  f   s     f

251
L F I L F L P M K D
b b b b b b e b e e
s             s   f

261
I Q S V D A S Y N R
b e e b e b b b b e
        f   s   s

271
I T I N N I D V E A
b b b e e e e e e e

281
I C H F P F S N F S
b b e b e b e e b b

291
F L N I S N N P I N
b b b b e e e e b e
    s s f f f f

301
S L E T V C L P A S
b b e e b b b e e b
  s           f

311
I T V I D L S F T N
b e b b e b b b b e
      s f s s   s

321
I S T I P A N F A K
b e e b e e e b b e
s     s

331
K L S K L E R M Y V
e b e e b e e b b b
          f

341
Q G N Q L I Y T V R
e e e e b b b e b e
  f f

351
P E N P S A T P R P
e e e e e e b e e e

361
P P G T V Q I S A I
e e e e b e b e b b

371
S L V R N Q A G T P
b b b e b e e e e e
s                 f

381
I E S L P E S V K H
b e e b e e e b e e
s   f   f

391
L K V S N C S I V E
b e b e e b b b b e
        f   s s s

401
L P E W F A N R M Q
b e e b b e e e b e
s f

411
E L L F L D L S S N
e b b b b e b e e e
            s f f f

421
R I S M L P D L P I
e b e e b e e b e e
          f

431
S L Q Q L D I S N S
e b e e b e b e e e
f         f   f f

441
D I K I I P P R F K
e b e e b e e e b e
          f

451
S L S N V T V F N I
e b e e b b b b e b
      f   s s     s

461
Q N N K L T E M H P
e e e e b e e b e e
    f f

471
E Y F P S T L T T C
e e b e e b b e e b
      f     s

481
D I S K N K L K V L
e e e e e e b e e b
f   f f f

491
S L T K A L E N L E
e b e e b b e e b e

501
S L N V S G N L I T
b b e b b e e b b e
    f   s   f

511
R L E P A C Q L P S
e b e b e e e e e b

521
L T N L D S S H N L
b e e b e b b e e b
        f   s f f

531
I S E L P D H L G Q
b e e b e e e b e e
  f   s

541
S L L M L K H F N L
b b e e b e e b e b
        s f     f

551
S G N K I S F L Q R
e e e e b b b b e e
f   f f s s   s f

561
G S L P A S L E E L
e e e e e b b b e b
f f f f   s s

571
D I S D N A I T T I
e b b e e b b b b b
f   s   f s s s s s

581
V Q D T F G Q L T S
b e e b b e e b b e
      s s     s

591
L S V L T V Q G K H
b e b b e b e e e e
s     s     f f f f
```

```
601                   611                   621                   631                   641
F F C N C D L Y W F   V N I Y I R N P H L   Q I N G K D D L R C   S F P P D R R G S L   V K S S N L T L L H
b b b e b e b b b b   b b b b b e e e e b   e b e e e e e b e b   e b e e e e e e e e   b e e b e b b b b b
s s s f s f s   s       s                         f           s       f       f f             f

651                   661                   671                   681                   691
C S L G I Q M A I T   A C M A I L V V L V   L T G L C W R F D G   L W Y V R M G W Y W   C M A K R R Q Y K K
b b b e b b b b b b   b b b b b b b b b b   b b b b b b b b e b   e b b b e b b b b b   b b b e e e e e e e
s         s     s s         s                     s s s     f       s s     s s s s s   s s s f f   f f   f

701                   711                   721                   731                   741
R P E N K P F D A F   I S Y S E H D A D W   T K E H L L K K L E   T D G F K I C Y H E   R D F K P G H P V L
e e e e e e b e b b   b b e b e e e b e b   b e e e b b e e b e   e e e b e b b b e e   e e e e e e e b b e
    f   f     f s s     s f s f   f s   s     f             s f             s s s f f   f f f   f f f s s f

751                   761                   771                   781                   791
G N I F Y C I E N S   H K V L F V L S P S   F V N S C W C Q Y E   L Y F A E H R V L D   E N Q D S L I M V V
b b b b b b b e e b   e e b b b b b b e b   b b e b b b b e b e   b b b b b b b b b e   e e e e b b b b b b
  s s s     s f f s   f f s   s s s s   s   s s f s s s s f s f   s   s s s s s s s     f f f f s s s s   s

801                   811                   821                   831                   841
L E D L P P D S V P   Q K F S K L R K L L   K R K T Y L K W S P   E E H K Q K I F W H   Q L A A V L K T T N
b e e b e e e e e e   e e b e e b e e b b   e e e e e b e e e e   e e e e e e b b b e   e b b e b b e e e e
s f f s f     f   f   f f s f f s f f s s     f f f f s f f f     f f f f f     s s     f s     s s   f   f

851                   861
E P L V R A E N G P   N E D V I E M E
e e b b e e e e e e   e e e e e e e e
  f                             f
```

## The conservation scale:

| ? | 1 | 2 | 3 | 4 | 5 | 6 | 7 | 8 | 9 |
|---|---|---|---|---|---|---|---|---|---|

**Variable** **Average** **Conserved**

e - An exposed residue according to the neural-network algorithm.

b - A buried residue according to the neural-network algorithm.

f - A predicted functional residue (highly conserved and exposed).

s - A predicted structural residue (highly conserved and buried).
