# Supplementary material for: Uncovering a 500 million year old history and evidence of pseudogenization for TLR15
Source: Front Immunol. 2022 Dec 20;13:1020601. doi: 10.3389/fimmu.2022.1020601 (PMC9808068; doi:10.3389/fimmu.2022.1020601)
Supplement: Supplementary file 1 [file DataSheet_1.zip › Data Sheet 1/Supplementary_Material.docx]

Supplementary Material

# Supplementary Tables

**Supplementary Table 1.** Genomic and transcriptomic resources used in bioinformatic searches for TLR15 gene.

**Supplementary Table 2.** Phylogenetic test of positive selection.

**Supplementary Table 3.** Phylogenetic test of negative selection.

**Supplementary Table 4.** Genomic location of the genes presented in Figure 3

# Supplementary Figures

**Supplementary Figure 1.** Amino acid alignment of TLR15. (*) represent Stop codons; (.) represent identity with the reference sequence (*Gallus gallus*); (−) represent indels; predicted signal peptides are highlighted in yellow; internal repeat regions are underlined; LRR are shaded in grey; C terminal is shaded in dark grey; TIR domain in light grey; the TM domain is boxed; the proline-rich loop in LRR9 is in a dashed box and the arrow identifies the Proline site that mediates MyD88 interaction. The species represented are, from top to bottom: Galga - *Gallus gallus* (chiken), Lepdi -  *Leptosomus discolor* (cuckoo roller), tauer - *Tauraco erythrolophus* (red-crested turaco), Gavga - *Gavialis gangeticus* (gharial), Cropo - *Crocodylus porosus* (Australian saltwater crocodile), Allsi - *Alligator sinensis* (Chinese alligator), Anoca - *Anolis carolinensis* (green anole), Promu - *Protobothrops mucrosquamatus* (brown spotted pit viper), Podmu - *Podarcis muralis* (common wall lizard), Gekja - *Gekko japonicus* (Schlegel's Japanese gecko), Proan - *Protopterus annectens* (West African lungfish), Neofo - *Neoceratodus forsteri* (Australian Lungfish), Calmi - *Callorhinchus milli* (Australian ghostshark), Hydco - *Hydrolagus colliei* (spotted ratfish), Hydmi - *Hydrolagus mirabilis* (large-eye rabbitfish) and Chiop - *Chimaera opalescens* (opal chimaera). Numbering is according to chicken TLR15, with signal peptide and indels (indicated as (-)) being included in the numbering.

**Supplementary Figure 2.** a. Nucleotide alignment of holocephalan TLR15 sequences. Below the nucleotide is indicated the corresponding translated amino acid. All nucleotide/amino acid agreements are highlighted. (*) represent early stop codons and are boxed in red; (−) represent indels. The one base pair deletion in Hydaf is boxed in black. The species represented are, from top to bottom: Calmi - *Callorhinchus milli* (Australian ghostshark), Hydco - *Hydrolagus colliei* (spotted ratfish), Hydmi - *Hydrolagus mirabilis* (large-eye rabbitfish) and Chiop - *Chimaera opalescens* (opal chimaera), Harra - *Harriotta raleighana* (narrownose chimaera) and Hydaf – *Hydrolagus affinis* (small-eyed rabbitfish). Numbering is according to Calmi TLR15, with signal peptide and indels (indicated as (-)) being included in the numbering. b. Detail of the region with the one base pair deletion in Hydaf. From the top is the Hydaf sequence retrieved from the genome, followed by the electropherograms from Sanger sequence with primers Forward and Reverse, respectively, and all the remaining sequences are from the SRA data obtained for Hydaf mapped to this particular region.

**Supplementary Figure 3.** Evolutionary conservation of amino acid positions displayed in the alignment of TLR15 sequences. The conservation scale is defined from the most variable (grade 1, colored turquoise) to the most conservative (grade 9, colored maroon) amino acid positions. The sequence of chicken TLR15 was used as query to show the nine-color conservation grades along the alignment.

**Supplementary Figure 4.** Comparison of the expected read counts between TLR2, TLR3 and TLR15 in *C. milii*.

**Supplementary Figure 5.** Comparison of the expected read counts between TLR2, TLR3 and TLR15 in *H. colliei*.

**Supplementary Figure 6.** SMART structures of vertebrates TLR15. Dark rectangles represent TM domain; green bands indicate LRRs involved in PAMP recognition (LRR-TYP represents typical LRRs, whereas LRR denotes LRR outliers); light blue circles represent C terminal LRR; pink boxes indicate low compositional complexity regions; and blue boxes numbered with RPT indicated internal repeat regions.

# Supplementary data

**Supplementary data 1.** Nucleotide sequences of the TLR15 gene for the species analyzed in this study.
