# Supplementary figures and images for: Uncovering a 500 million year old history and evidence of pseudogenization for TLR15
Source: Front Immunol. 2022 Dec 20;13:1020601. doi: 10.3389/fimmu.2022.1020601 (PMC9808068; doi:10.3389/fimmu.2022.1020601)

a

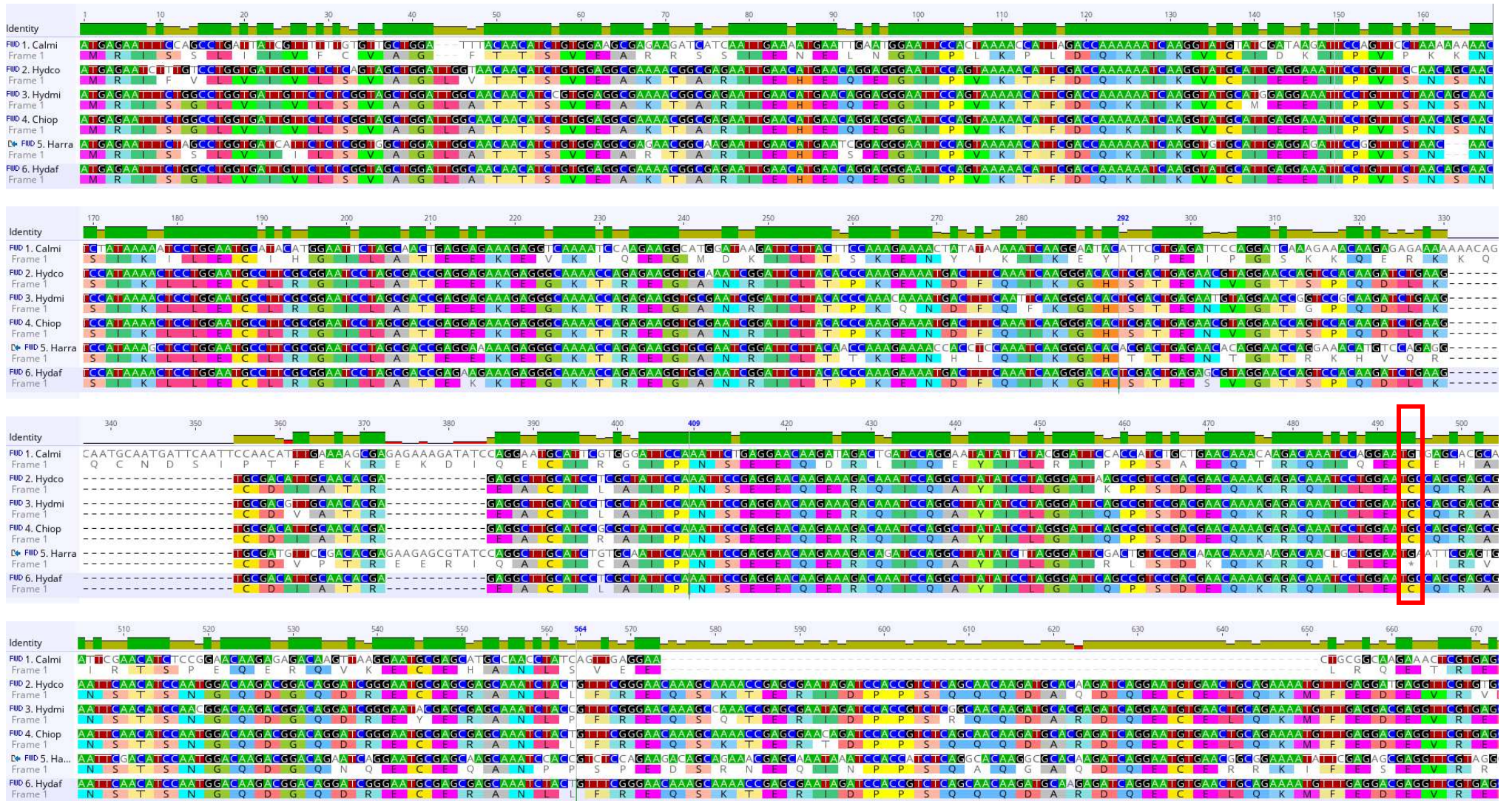

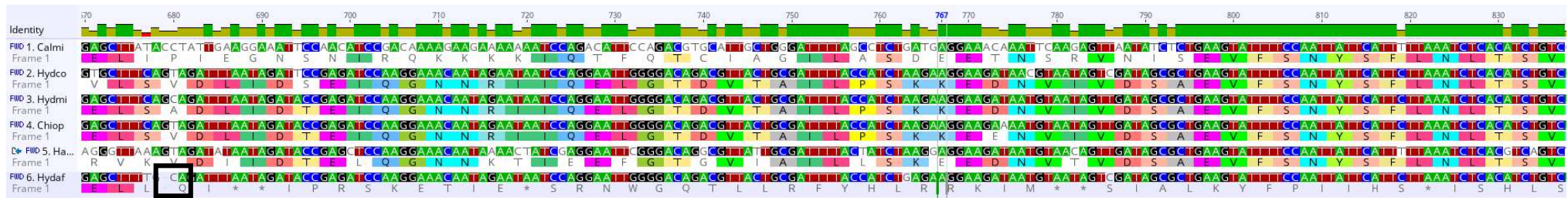

b

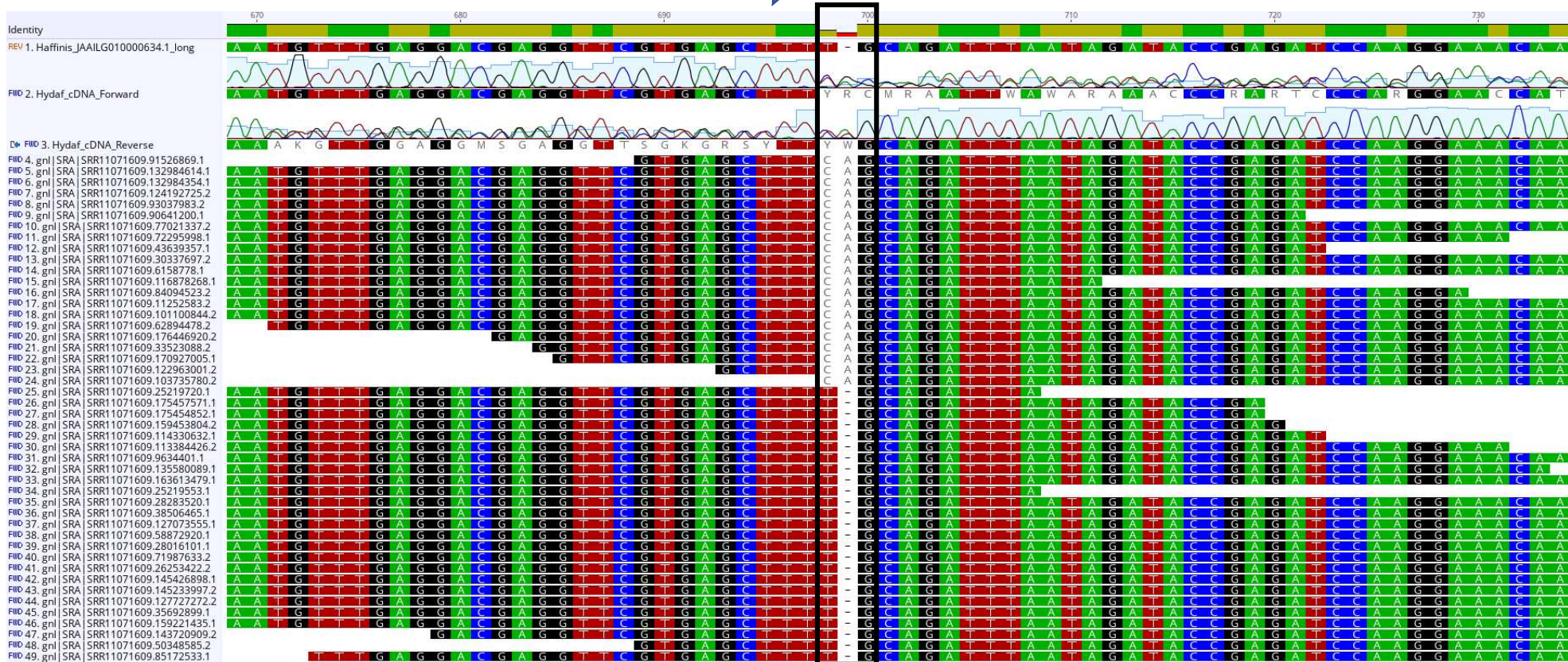

Supplement: Supplementary file 1 [file DataSheet_1.zip › Data Sheet 1/Supplementary Figure 2.pdf]

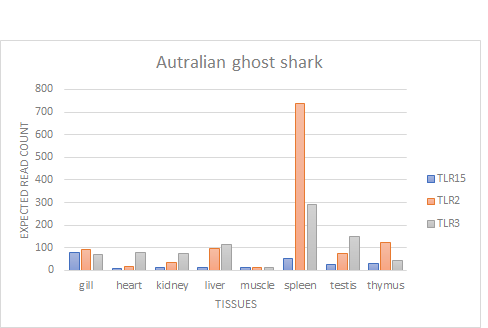

Supplement: Supplementary file 1 [file DataSheet_1.zip › Data Sheet 1/Supplementary Figure 4.tif]

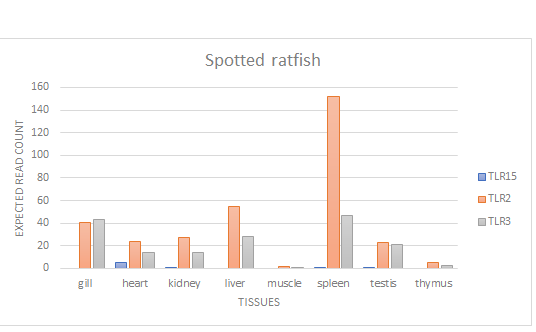

Supplement: Supplementary file 1 [file DataSheet_1.zip › Data Sheet 1/Supplementary Figure 5.tif]

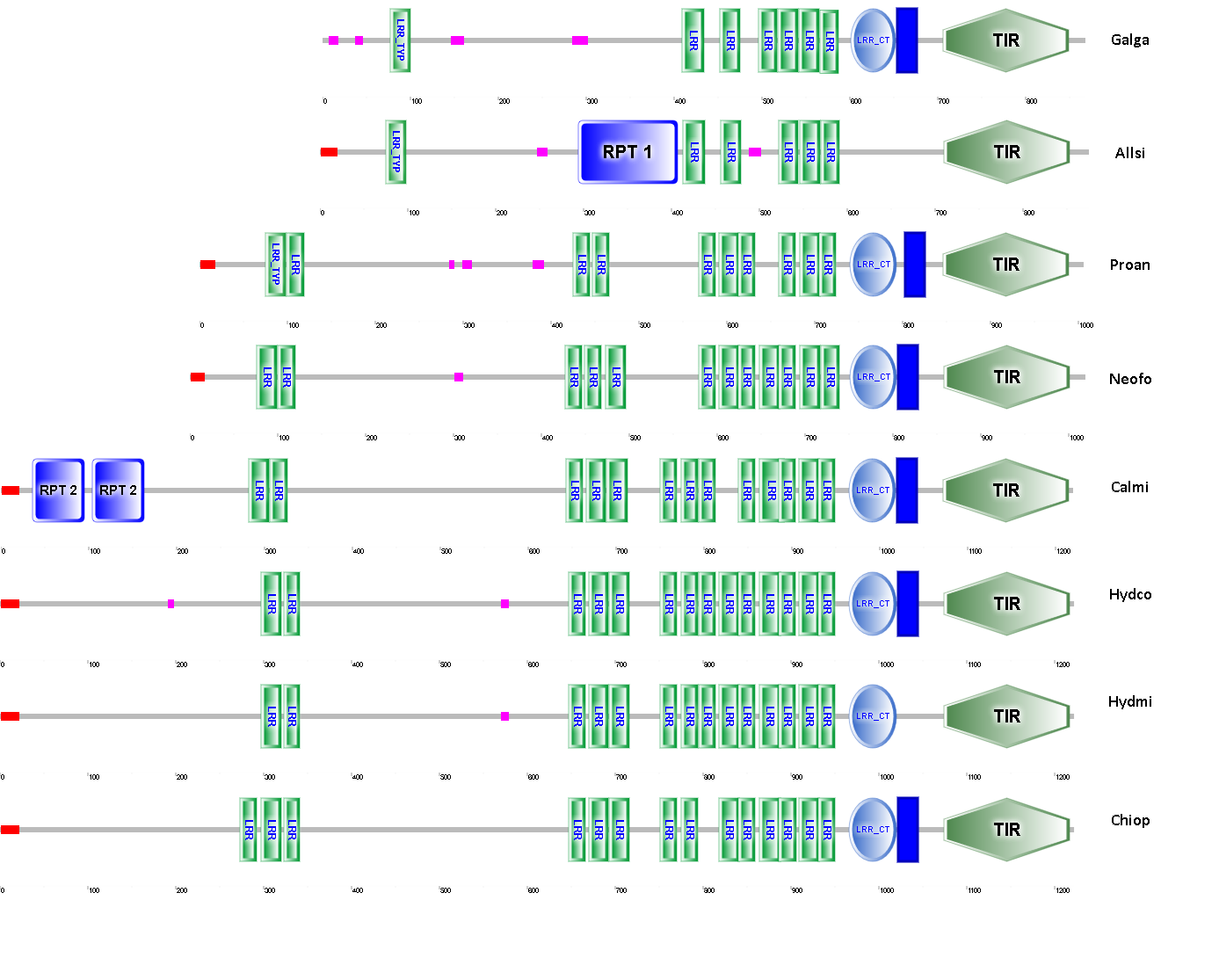

Supplement: Supplementary file 1 [file DataSheet_1.zip › Data Sheet 1/Supplementary Figure 6.tif]
